# Stress hyperglycemia and poor outcomes in patients with ST-elevation myocardial infarction: a systematic review and meta-analysis

**DOI:** 10.3389/fcvm.2024.1303685

**Published:** 2024-03-11

**Authors:** Abdul Hakim Alkatiri, Nurul Qalby, Idar Mappangara, Ahmad Taufik F. Zainal, Maarten J. Cramer, Pieter A. Doevendans, Andriany Qanitha

**Affiliations:** ^1^ Department of Cardiology and Vascular Medicine, Faculty of Medicine, Hasanuddin University, Makassar, Indonesia; ^2^ Makassar Cardiac Center, Dr. Wahidin Sudirohusodo General Teaching Hospital, Makassar, Indonesia; ^3^ Department of Public Health and Community Medicine, Faculty of Medicine, Hasanuddin University, Makassar, Indonesia; ^4^ Heart and Lung Division, Department of Cardiology, University Medical Center Utrecht, Utrecht, Netherlands; ^5^ Faculty of Medicine, Hasanuddin University, Makassar, Indonesia; ^6^ Netherlands Heart Institute, Utrecht, Netherlands; ^7^ Department of Physiology, Faculty of Medicine, Hasanuddin University, Makassar, Indonesia; ^8^ Doctoral Study Program, Faculty of Medicine, Hasanuddin University, Makassar, Indonesia

**Keywords:** adverse outcomes, hyperglycemia, MACCE, mortality, STEMI, revascularization

## Abstract

**Background:**

Hyperglycemia, characterized by elevated blood glucose levels, is frequently observed in patients with acute coronary syndrome, including ST-elevation myocardial infarction (STEMI). There are conflicting sources regarding the relationship between hyperglycemia and outcomes in STEMI patients. We aimed to compile evidence to assess the association between hyperglycemia and adverse outcomes.

**Methods:**

We conducted a comprehensive search for articles on PubMed and Embase using search strategies which yielded 4,061 articles. After full-text screening, 66 articles were included for systematic review, and 62 articles were further selected for meta-analysis.

**Results:**

The 66 included articles spanned the years 2005–2023. Of these, 45 articles reported admission blood glucose, 13 articles used HbA1c, and 7 articles studied fasting blood glucose. Most studies defined STEMI with primary PCI as their inclusion criteria. Mortality was the most often outcome reported related to hyperglycemia. Overall, 55 (83.3%) studies were at low risk of bias. Both admission and fasting blood glucose were significantly related to short- and long-term mortality after STEMI, with a pooled risk ratio (RR) of 3.02 (95%CI: 2.65–3.45) and 4.47 (95% CI: 2.54–7.87), respectively. HbA1c showed substantial association with long-term mortality (HR 1.69, 95% CI: 1.31–2.18)) with a pooled RR of 1.58 (95% CI 1.26–1.97). In subsequent analyses, admission hyperglycemia was associated with an increased risk of reinfarction (pooled RR 1.69, 95% CI 1.31–2.17), heart failure (pooled RR 1.56, 95% CI: 1.37–1.77), cardiogenic shock (pooled RR 3.68, 95% CI 2.65–5.11), repeat PCI or stent thrombosis (pooled RR 1.99, 95% CI 1.21–3.28), and composite major adverse cardiac and cerebrovascular events (MACCE) (pooled RR 1.99, 95% CI: 1.54–2.58).

**Conclusions:**

Our study demonstrated that hyperglycemia has a strong association with poor outcomes after STEMI. Admission and fasting blood glucose are predictors for short-term outcomes, while HbA1c is more appropriate for predicting longer-term outcomes in STEMI patients.

**Systematic Review Registration:**

PROSPERO 2021 (CRD42021292985).

## Introduction

Coronary heart disease (CHD) is a leading cause of morbidity and mortality worldwide. The most common type of CHD is acute myocardial infarction (MI). Each year, it is reported that at least 15% of deaths are caused by MI, with the majority presenting as ST-elevation myocardial infarction (STEMI) (1). To date, there is increasing evidence reporting an association between the incidence of hyperglycemia and poor outcomes in STEMI patients. Several studies have demonstrated that hyperglycemia significantly increased mortality in patients with STEMI, both during the hospital stay and several days or months after the first diagnosis (2–4). Other studies have also reported a significant association between hyperglycemia in STEMI patients and reperfusion failure (5–7).

Hyperglycemia is a frequent condition observed in patients with acute MI, even in the absence of a history of diabetes mellitus. The pathological stress response, a series of neurohormonal reactions activated during acute MI, leads to excessive sympathetic nerve activation, resulting in elevated blood sugar levels (5, 8, 9). However, the precise mechanism by which hyperglycemia contributes to poor outcomes in STEMI patients is currently not clearly understood. Therefore, the primary objective of this systematic review is to summarize the evidence regarding the association between stress-induced hyperglycemia and adverse clinical outcomes in patients with ST-elevation Myocardial Infarction (STEMI). We explicitly formulate the review questions as follows: (1) Does hyperglycemia increase the risk of mortality and major adverse cardiac and cerebrovascular events (MACCE) in patients with STEMI? (2) What is the optimal cutoff point for diagnosing hyperglycemia in critically ill patients, especially those with STEMI?

## Methods search strategy

This review was registered in The International Prospective Register of Systematic Reviews (PROSPERO) database (registration number CRD42021292985) and conducted in accordance with the PRISMA guidelines (10). The literature search was performed in December 2021 and additionally in January 2024 to cover the recently updated literature. We utilized the following databases: PubMed (Medline), EMBASE (Ovid), and Web of Science. We employed a combination of MeSH terms and free-text words to identify relevant articles. Additionally, we screened the reference lists of published reviews to identify any additional relevant studies. Detailed information regarding the search strategy is presented below:

#1. Hyperglycemia OR stress hyperglycemia OR high blood sugar OR blood glucose OR diabetes
#2. Coronary heart disease OR Acute coronary syndrome OR acute myocardial infarction OR ST-elevation myocardial infarction.
#3. Prognosis OR outcomes OR adverse events OR MACE OR MACCE OR mortality OR death OR cardiovascular death OR re-hospitalization OR recurrent MI OR re-infarction OR recurrent HF OR stent thrombosis OR repeat PCI OR emergency CABG OR recurrent stroke.

#1 AND #2 AND #3 AND #4 NOT animal.

Our detailed search strategies were provided as (Supplementary Table S1).

### Eligibility criteria

Studies meeting the following criteria were included in this systematic review and meta-analysis:

(1) Cohort (prospective or retrospective) or case-control studies conducted in STEMI patients.
(2) Outcomes were clearly defined as follows: mortality (in-hospital, at 30 days, at 6 months, or >6 months after discharge), recurrent MI, stent thrombosis or repeat PCI, emergency CABG, development of heart failure (acute pulmonary edema), cardiogenic shock, stroke, and longer hospitalization.
(3) Blood glucose or hyperglycemia was quantified (at admission, within 24 h, within >24 h of admission, or at another specific time during hospitalization).
(4) Sufficient data on clinical outcomes (mortality, MACCE, or other complications as previously stated), including relative risks (RR), odds risks (OR), Hazard Ratio (HR), and their corresponding 95% confidence interval (CI).

In the case of serial or updated articles based on the same study, only the last published report was selected for analysis, while previous reports were considered supplementary for missing data where applicable. Only full articles in English were included. A manual search and snowballing method for additional relevant studies using references from retrieved articles were also completed. Articles were searched independently by two investigators (AQ and NQ), and all included abstracts were exclusively collected using the Rayyan—Intelligent Systematic Review application (https://www.rayyan.ai) for further screening.

**Figure 1:**
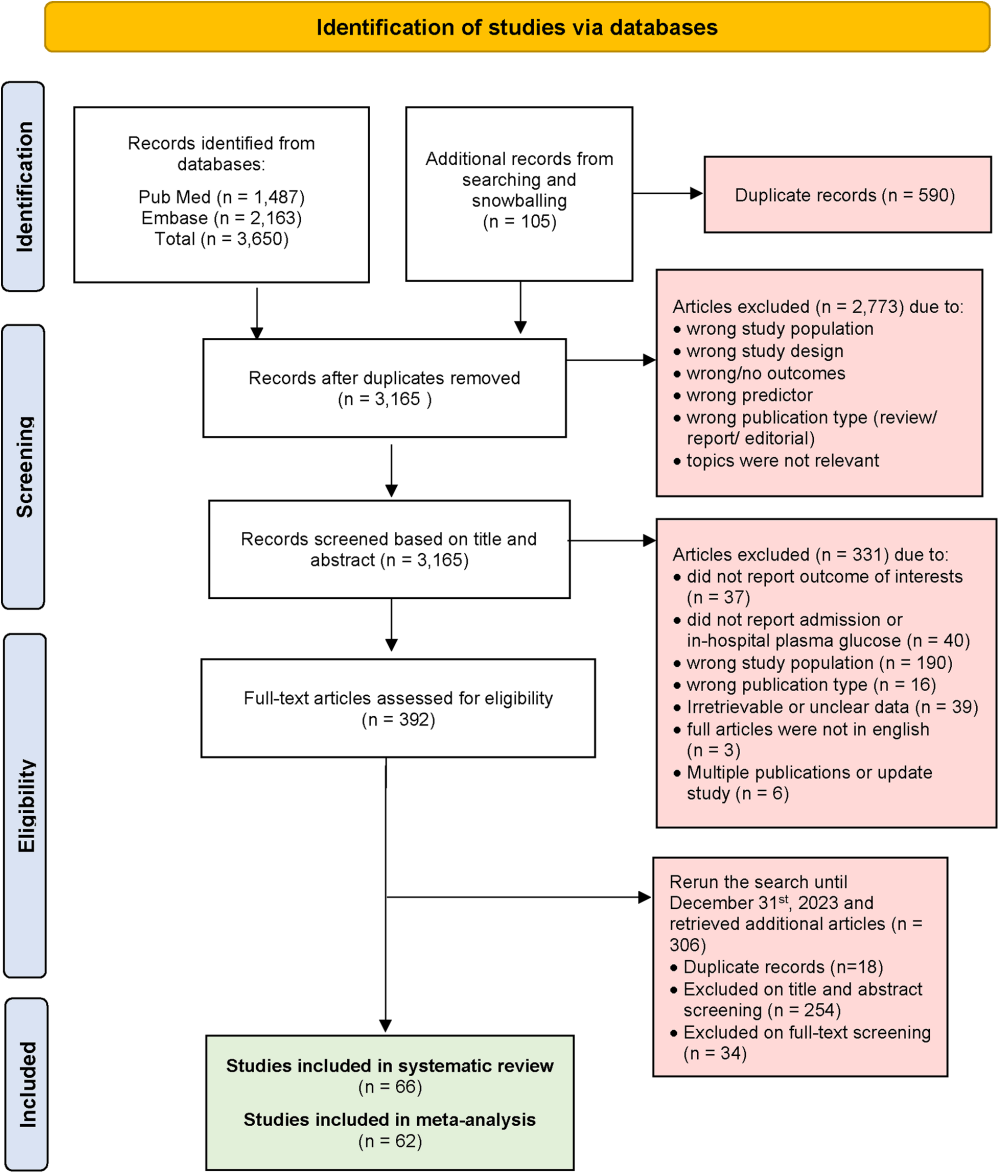
PRISMA flow diagram of selected studies.

Generally, we excluded studies if the abstract or full-text paper in English were not accessible. Studies were excluded if they lacked sufficient information to calculate RR. Additionally, cohort studies that did not explicitly report the proportion of patients followed up, blood glucose measurement, and those that followed up less than 70% of patients were also excluded. Detailed reasons for study exclusion are clearly reported in the PRISMA flow chart (see Figure 1).

### Study selection

Following the literature search, AQ and NQ independently screened the titles and abstracts. Any disagreements were resolved through mutual consensus. Literature studies that met the eligibility criteria were included, while those that did not meet the criteria were excluded with specific reasons provided. Conflicts in selecting the studies were discussed, and additional judgment was provided by the third reviewer (AHA) until a consensus was reached.

### Data extraction

Three investigators (AQ, NQ, and AT) independently screened the full-text article, performed data extraction, and assessed the risk of bias in each individual study. Any discrepancies in data were resolved by reviewing the primary data from the original articles. We employed a standardized data extraction approach using a Google form and Microsoft Excel. The extracted variables included the first author's name and year of publication, study design, country, recruitment period, follow-up duration, study population, sample size, male gender, age, hyperglycemia definition and cut off, measured outcomes, and conclusions (OR, RR or HR).

The extracted outcome data, including mortality and MACCE, were analyzed for both hyperglycemic and non-hyperglycemic group. In cases where the included literature study had incomplete or inaccessible data, the study was excluded with the agreement of the reviewers.

### Definition

ST-elevation myocardial infarction (STEMI*)* was defined as the presence of at least two contiguous leads with ST-segment elevation ≥2.5 mm in men under 40 years, ≥ 2 mm in men aged 40 years or older, or ≥1.5 mm in women in leads V2-V3 and/or ≥1 mm in the other leads (11). Patients were classified as having diabetes if they had a reported history of diabetes. Glycated haemoglobin (HbA1C) and blood glucose levels on admission were not integrated because HbA1C was not measured in all studies, and stress glucose concentrations corresponding to diabetes cutoff values (i.e., fasting plasma glucose of 7.0 mmol/L or 2 h glucose 11.1 mmol/L on a 75 g oral glucose tolerance test) were undefined. Hyperglycemia was defined according to the definitions used in individual studies, resulting in varying threshold glucose concentrations to define hyperglycemia across studies.

### Quality assessment

To assess the individual studies, we evaluated the risk of bias using the QUIPS (Quality in Prognosis Study) tool (12). This tool assesses six domains: study participation, study attrition (for cohorts), prognostic factor measurement, outcome measurement, study confounding, and statistical analysis and reporting. Each domain was graded as low, moderate, or high risk of bias, with specific appraisal criteria for each domain. The overall risk of bias for each individual study was graded as follows: high risk of bias (if ≥4 of 6 domains showed high risks), medium risk of bias (if ≥4 of 6 domains showed medium risk), and low risk of bias (if ≥4 of 6 domains showed low risk).

### Statistical analysis

We conducted a meta-analysis of the included studies using a random-effects model. Point estimates of RR, OR, or HR, along with their respective 95% confidence intervals (CIs), were pooled from each individual study to assess the association between hyperglycemia and clinical outcomes in STEMI patients. Heterogeneity in effect size estimates across these studies was examined using the Chi- squared test and quantified using the *I*^2^ statistic. The *I*^2^ statistic, with values ranging from 0 to 100% was classified as follows: low heterogeneity (if *I*^2^ < 25%), moderate heterogeneity (if *I*^2^ = 25%–49%), and substantial heterogeneity (if *I*^2^ > 50%). Publication bias was assessed using a funnel plot and the Egger's regression test, with *p* < 0.05 considered statistically significant. All data analyses were performed using Review Manager (RevMan) 5.4, SPSS ver. 26 for mac, and RStudio veer. 1.4.1564 for Windows.

## Results

### Study selection

At initial systematic searching, we retrieved a total of 3,650 articles from PubMed and Embase using the search strategy. An additional 105 articles were identified through snowballing of selected articles and previous systematic reviews. After excluding 590 duplicate articles, 3,165 articles were screened based on their titles and abstracts. This process left us with 392 articles for full-text review. Then we extended our search to include studies published until 31st December 2023, and found 306 articles with 18 duplicates. Articles were excluded if they did not report blood glucose level parameters or outcomes of interest. Additionally, articles were excluded if data from STEMI patients were not extractable. Ultimately, 66 articles were included for systematic review, and after data assessment, 62 articles were included for meta-analysis. A visual representation of the screening process is provided in Figure 1 (PRISMA flow diagram).

### Study characteristics

We summarized the 66 included articles in Table 1. The publication years of the included studies ranged from 2005 to 2023. In our additional literature searching from January 2022 to December 2023, we incorporated five articles that were employed in both systematic reviews and meta-analyses (*n* = 66). Among these, 46 articles were prospective cohort studies, 19 were retrospective cohort studies, and 1 was a case-control study. Hyperglycemia was defined using various parameters: 47 articles used admission blood glucose, 13 used HbA1c, and 7 used fasting blood glucose. Other parameters such as mean and peak glucose, as well as triglyceride-glucose (TyG) index, were also reported in some studies, however we did not include these parameters in our meta-analysis. Most articles reported mortality at different time points (i.e., in-hospital, 30-day, 6-month, >6-month mortality). Other outcomes analyzed in this review included recurrent myocardial infarction, heart failure, stroke, cardiogenic shock, repeat PCI, emergency CABG, and composite MACCE.

**Table 1:** Studies included in systematic review (*n* = 66) and meta-analysis (*n* = 62).

No	Author, year of publication	Study design	Study population	Period of recruitment & follow-up duration	Total sample size	Male, *n* (%) Age (years)	Hyperglycaemia definition	Hyperglycaemia cut-off	DM *n* (%)	STEMI treatment	Outcomes, events/total	Conclusions (RR/HR/OR, 95%CI) *p*-value
1	Aggarwal et al. 2016 (13)	Cohort retrospective	STEMI patients undergoing primary PCI without a history of diabetes mellitus	2005–2012 (3 years)	1,686	1,137 (67.4) Age 60.4 ± 13.2 years	HbA1c ≥ 6.5% and no history of DM or DM therapy	HbA1c ≥ 6.5%	0 (0)	Primary or rescue PCI	In-hospital mortality HG (14/118), non-HG (51/1,163) 3 -year mortality HG (28/118), non-HG (136/1,163)	Patients with HbA1c >= 6.5% associated with increased 3-year mortality, with adjusted HR 1.7 (1.0–3.0), *p* = NA
2	Ahmad et al. 2012 (14)	Case-control	STEMI patients	2010–2012 30 days	754	512 (67.91) Age 51.97 ± 8.36 years	HbA1c > 6.5%	HbA1c > 6.5%	352 (46.69)	NA	30-day mortality HG (72/352), non-HG (45/402)	Higher levels of HbA1C in diabetic as well as non- diabetic patients increase the risk of short-term mortality after acute myocardial infarction
3	Chang et al. 2013 (15)	Cohort prospective	Primary STEMI	April-December 2005 Average 89 ± 20 months	83	66 (79.52) Age 59.2 ± 10.7 years	Admission blood glucose level ≥ 200 mg/dl	≥200 mg/dl	20 (24.10)	Primary or rescue PCI	8-year mortality HG (5/24), non-HG (3/56) In-stent restenosis HG (2/24), non-HG (3/56)	Patients with high glucose levels at ad- mission glucose had higher mortality on long-term follow up compared to those with normal glucose levels at admission
4	Chen et al. 2014 (3)	Cohort prospective	STEMI patients undergoing primary PCI	1992–2008 (65.9 ± 3.2 months)	959	787 (82.1) Age 61.5 ± 12.6 years	Admission glucose level ≥ 190 mg/dl	Categorized into five groups based on admission glucose levels of <100, 100–139, 140–189, 190–249 and ≥250 mg/dl.	306 (31.9)	Primary PCI	In-hospital Mortality HG (52/305), non-HG (28/654) Cardiogenic shock HG (100/305); non-HG (106/654) Length of ICU stay HG (4.9 ± 6.0 days); non-HG (2.9 ± 1.8 days) Length of hospital stay HG (10.0 ± 8.7 days); non-HG (7.6 ± 6.3 days) Reinfarction HG (14/305); non-HG (16/654) HF hospitalization HG (30/305); non-HG (37/654) Long-term mortality HG (35/305); non-HG (27/654) MACE HG (62/305); non-HG (74/654)	Patients with Glucose ≥ 190 mg/dl In-hospital mortality OR = 2.74 (95%CI 1.4–5.5), *p* = 0.004 Mortality at follow up OR = 2.52 (95% CI)1.2–5.1), *p* = 0.01
5	Chioncel et al. 2009 (16)	Cohort prospective	Non-diabetic patients with acute STEMI	Between 7 January 2006 and 30 June 2007 (30 days)	128	137 (80.6) Age 60.1 years	Admission blood glucose level > 200 mg/dl	Group 1: <140 mg/dl Group 2: 140–200 mg/dl Group 3: >200 mg/dl	42 (24.7)	Fibrinolysis	30-day mortality: HG (7/37), non-HG (11/91) 30-day mortality: HG non-DM (7/37), HG-DM (8/42) In-hospital HF: HG (2/37), non-HG (5/91) In-hospital HF: HG non-DM (2/37), HG-DM (4/42) LVEF: 49.5% (group 1) vs. 43.4% (group 2 vs. 38.5% (group 3 vs. 37.2% (diabetes group), *p* < 0.05	Admission glucose level: death (175 ± 55 mg/dl) vs. survive (146 ± 36 mg/dl)
6	Cicek et al. 2011 (17)	Cohort prospective	STEMI patients who were admitted to the emergency department within 12 h and underwent urgent cardiac catheterization procedures	December 2009—June 2010″ During hospitalization	374	318 (85.0) Age 55.9 ± 12.6 years	HbA1c ≥ 6.5%	HbA1c ≥ 6.5%	66 (17.65)	Primary or rescue PCI	In-hospital mortality HG (9/82), non-HG (6/292) Recurrent MI HG (1/82), non-HG (5/292) Stroke HG (1/82), non-HG (0/292) Cardiogenic shock HG (8/82), non-HG (6/292) Acute stent thrombosis HG (1/82), non-HG (7/292)	HbA1c ≥ 6.5% In-hospital mortality OR 1.412 (1.031–1.935), *p* = 0.03
7	Demarchi et al. 2021 (18)	Cohort prospective	STEMI patients undergoing primary PCI	2005–2017 1 year	2,958	2,248 (76) Age 62 ± 13 years	Admission plasma glucose >198 mg/dl	>198 mg/dl	244 (8.25) on hyperglycaemic group	Primary or rescue PCI	1-year mortality HG (86/488), non-HG (129/2,470)	Admission plasma glucose to 1 year mortality HR 1.9, (1.5–2.9), *p* = 0.001
8	De Monte et al. 2008 (19)	Cohort prospective	STEMI undergo PCI	2004–2006 30 days	184	NA Age 65 ± 13 years	Admission plasma glucose >110 mg/dl.	>110 mg/dl.	58 (31)	Primary or rescue PCI	30-days mortality HG (10/104), non-HG (1/22) Heart Failure HG (22/104), non-HG (4/22)	The hyperglycemic non-diabetic group had a double risk of death compared to normoglycemic one.
9	Dharma et al. 2019 (20)	Cohort retrospective	STEMI patients who underwent primary PCI	2014–2016 1 year	856	751 (87.7) Age 55.7 (49.1–62.4) years	Admission blood glucose ≥169 mg/dl	≥169 mg/dl	231 (26.99)	Primary or rescue PCI	In-hospital mortality HG (29/307), nonHG (17/549) 1-year mortality HG (50/307), nonHG (33/549)	Admission plasma glucose >=169 mg/dl 1 year mortality, HR 2.0 (1.13–3.53), *p* = 0.01
10	Dong-Bao Li et al. 2011 (21)	Cohort prospective	ST-Elevation AMI patients, who were admitted within 12–24 h after the onset of symptoms, underwent an emergency PCI or drug therapy or thrombolysis	From April 1995 to May 2005 (During hospitalisation)	1,137	821 (72.2) Age 62.6 ± 11.5 years	Based on nonfasting glucose level on admission: Hypoglycemia (<5 mmol/L) Euglycemia (5–7 mmol/L) Mild hyperglycemia (7–9 mmol/L) Moderate (9–11 mmol/L) Severe (>11 mmol/L)	126 mg/dl	246 (21.6)	Both fibrinolysis and PCI	In-hospital mortality: HG (92/544), non-HG (48/593) Hypoglycaemia 19 (10.4%) Euglycemia 29 (7.1%) Mild Hyperglycemia 18 (7.8%) Moderate Hyperglycemia 21 (18.6%) Severe Hyperglycemia 53 (26.4%)	Elevated admission glucose levels are associated with an increased risk of life-threatening complications in ST-Elevation AMI patients (*p* < 0,05)
11	Eitel et al. 2012 (22)	Cohort prospective	Patients with STEMI undergoing primary PCI with symptom onset <12 h and had cardiovascular magnetic resonance imaging (CMRI)	From February 2006 to August 2008 [19 (IQR 12–26) months]	411	306 (75.0) Age 65 (55–73) years	Hyperglycemia if admission blood glucose ≥7.8 to 11.0 mmol/L, and severe hyperglycemia ≥11.1 mmol/L	Normoglycemia <7.8 mmol/L (<140 mg/dl), hyperglycemia ≥7.8 to 11.0 mmol/L (≥140–199 mg/dl) severe hyperglycemia ≥11.1 mmol/L (≥ 200 mg/dl)	88 (21.4)	Primary PCI	LVEF (%) in non-DM HG [44.9 (36.7–55.5)], non HG [50.4 (42.9–58.4)] LVEF (%) in DM HG [47.1 (33.1–56.8)], non HG [48.4 (42.9–58.9)]	MACE in overall cohort HR 2.6 [CI, 1.6–4.4]; *P* < 0.001 MACE in non-DM HR 2.2 [CI 1.6–2.9], *P* = 0.007 MACE in DM HR 1.9 [CI, 1.1–3.4]; *P* = 0.05 MACE (admission glucose) HR 1.09 (1.03–1.17), *P* = 0.03
12	Ekmekci et al. 2013 (23)	Cohort retrospective	Patients who were ≥ 65 years and underwent primary percutaneous coronary intervention (PCI) for STEMI	October 2003 to March 2008 (mean 18.8 months)	677	454 (67.1) Age 72.1 ± 5.4 years	Admission blood glucose > 168 mg/dl	168 mg/dl	122 (18.0)	Primary PCI	In-hospital mortality HG (41/220), non-HG (23/457) Mortality >6 months HG (35/175), non-HG (41/426) Long-term stroke HG (2/175), non-HG (5/426) Long -term HF HG (26/175), no-HG (50/426) Long-term recurrent MI HG (24/175), non-HG (26/426)	In-hospital MACE with admission blood glucose(mg/dl), OR 1.009 (1.007–1.012) *p* < 0.001 (univariate)
13	Ekmekci et al. 2014 (24)	Cohort prospective	All consecutive patients admitted to the CCU with a diagnosis of acute STEMI within 12 h of onset, and treated with primary PCI	N/A (During coronary care and in hospital stay)	503	442 (87.9) Age 55.2 ± 12.5 years	Admission glucose >145 mg/dl	Tertile I: glucose <118 mg/dl; tertile II: glucose 118 to 145 mg/dl; and tertile III: glucose >145 mg/dl	0 (0)	Primary PCI	In-hospital mortality HG (9/169), non-HG (2/334) Cardiogenic shock HG (17/169), non-HG (8/334) Reinfarction HG (8/169), non-HG (5/334) Target vessel revascularization (Re-PCI) HG (7/169), non-HG (4/334) MACE HG (17/169), non-HG (7/334)	Admission glucose has an association with in-hospital MACE with adjusted OR 1.009 (95%CI 1.003–1.015), *p* = 0.01 Hyperglycemia at admission associated with in-hospital MACE: adjusted OR 9.55 (95%CI 1.99–46.5), *p* = 0.01
14	El-sherbiny et al. 2015 (25)	Cohort prospective	Nondiabetic acute STEMI candidate for reperfusion	January to December 2013 (6 months)	60	48 (80.0) Age 57.9 ± 8.9 years	Level of HbA1c with cut-off 6.5% as a diagnostic criteria of diabetes mellitus	HbA1c ≥ 6.5%	0 (0)	Primary PCI or thrombolytic	Reinfarction HG (2/27); non-HG (3/33) 6-month Mortality HG (2/27), non-HG (8/33) Heart failure HG (1/27); non-HG (3/33)	Admission higher HbA1c level in nondiabetic patients presented by acute STEMI is associated with more severe CAD, lower rate of complete revascularization TIMI 3, and higher incidence of adverse cardiac events and mortality.
15	Ergelen et al. 2010 (26)	Cohort retrospective	STEMI patients undergoing primary PCI	2003–2008 Median 21 months	2482	2,064 (83.2) Age 56.5 ± 11.9 years	Admission blood glucose ≥200 mg/dl	≥200 mg/dl	612 (24.7)	Primary or rescue PCI	In-hospital mortality HG (37/405), non-HG (33/2,077) Composite MACE HG (59/405), non-HG (95/2,077)	Long term cardiovascular mortality NDH (OR 3.04, 95% CI 1.06–8.73; *p* = 0.03) and DH (OR 2.3, 95% CI 1.29–4.09; *p* = 0.005)
16	Ferreira et al. 2021 (27)	Cohort retrospective	Primary STEMI patients	2006–2017 median 5.6 years (IQR 4.3)	1,234/2,768	1,888 (68.2) Age 68 ± 13 years	without DM Admission blood glucose >143 mg/dl) with DM Admission blood glucose > 213 mg/dl	without DM Admission blood glucose >143 mg/dl) with DM Admission blood glucose > 213 mg/dl	382 (24.91)	OMT based on ESC guideline	Without DM 2.5-year mortality HG (47/279), non-HG (51/573) 5-year mortality HG (72/279), non-HG (93/573) 7.5-year mortality HG (90/279), non-HG (117/573) 10-year mortality HG (104/279), non-HG (138/573) With DM 2.5-year mortality HG (29/159), non-HG (43/223) 5-year mortality HG (48/159), non-HG (73/223) 7.5-year mortality HG (60/159), non-HG (89/223) 10-year mortality HG (68/159), non-HG (100/223)	Hyperglycaemia in DM patients compared with normal glycaemia in non-DM patients increased mortality with HR 2.132, 95% CI 1.226–3.710, *p* = 0.007
17	Garadah et al. 2009 (28)	Cohort prospective	Patients with ACS	From January to December 2005 (During first week of hospitalization)	285	182 (63.9) Age 58.3 ± 15.9 years	Stress hyperglycemia was arbitrarily defined as admission glucose levels >7 mmol/L	Group 1 (control group): < 7 mmol/L (<126 mg/dl) Group 2: 7− ≤ 15 mmol/L (126–270 mg/dl); Group 3: > 15 mmol/L (>270 mg/dl)	84 (29.5)	Thrombolytic therapy in STEMI patients and other medications	In-hospital mortality HG (23/173), non-HG (7/112) More 2 MACCE HG (75/173), non-HG (12/112)	Death (admission glucose): OR 2.8 (95% CI 1.7–11.3), *p* = 0.03 Death (HbA1c): OR 1.4 (1.05–1.9), *p* = 0.04 Death (stress HG-DM): OR 3.3 (1.09–10.98), *p* = 0.04 Death (stress HG-nonDM): OR 3.2 (1.09–10.98), *p* = 0.03
18	Gasior et al. 2008 (29)	Cohort prospective	Acute STEMI patients referred for urgent invasive diagnostics with the intention of performing PCI	N/A 1 year	1,310	879 (86.5) Age 58 ± 10.6 years	Admission blood glucose level ≥ 7.8 mmol/L (140 mg/dl) was considered as hyperglycemic	Admission blood glucose ≥ 140 mg/dl	352 (26.9)	Primary or rescue PCI	In-hospital death HG (37/667), non-HG (8/643) In-hospital stroke HG (14/667), non-HG (5/643) 1-year stroke HG (13/667), non-HG (3/643) 1-year reinfarction HG (41/667), non-HG (35/643) 1-year death HG (81/667), non-HG (28/643) 1-year MACE HG (124/667), non-HG (63/643)	1-year mortality non-DM group HR = 1.09 (1.01–1.17)
19	Ghaffari et al. 2015 (30)	Cohort prospective	nondiabetic patients with STEMI	2012–2013 1 year	290	109 (62.4) Age 58.6 ± 13.2 years	Admission HbA1c > 5.8%	HbA1c > 5.8%	0 (0)	Fibrinolysis or PCI	In-hospital mortality HG (2/142), non-HG (2/148) 1-year mortality HG (11/142), non-HG (4/148) Stroke HG (0/142), non-HG (2/148) Recurrent MI HG (26/142), non-HG (15/148)	HbA1c > 5.8% in nondiabetic patients with STEMI was associated with severe CAD and multivessel involvement of the coronary arteries.
20	Ghariani et al. 2022 (31)	Cohort retrospective	Patients with STEMI and treated in the cardiology department of Farhat Hached university hospital center with urgent PCI (primary PCI or rescue PCI)	From January 2016 to December 2019 (12 months follow up)	225	167 (74.2) 61.1 ± 11.8	Admission blood glucose >180 mg/dl	>180 mg/dl	104 (46.2)	Primary or rescue PCI	Inhospital mortality HG (13/77), nonHG (5/148)	N/A
21	Gierach et al. 2016 (32)	Cohort prospective	Patients with first STEMI treated with CGA and primary PCI, without diabetes.	N/A 6 months	52/186	43 (82.70) Age 53.8 ± 7.85 years	Admission blood glucose level ≥ 148 mg/dl or 7.1 mmol/L	Admission blood glucose level ≥ 148 mg/dl	0 (0)	CAG and primary PCI	6-month mortality HG(0/26), non-HG (0/26) 6-month recurrent MI, HG(1/26), non-HG (0/26) 6-month stent thrombosis, HG (6/23), non-HG (6/23) 6-month acute HF (LVEF < 45%) HG (14/26), non-HG (6/26) 6-month CHF (LVEF < 45%) HG (7/26), non-HG (2/26) 6-month CVD hospitalisation HG (1/26), non-HG (2/26)	Multivariable analysis: higher admission glucose associated with lower LVEF in acute and 6-month follow up (*p* = 0.047).
22	Hoebers et al. 2012 (33)	Cohort prospective	Patients underwent primary PCI for STEMI	From March 2005 to December 2007 (3 years)	1,646	1,170 (71.1) Age 62 (49–78) years	Admission blood glucose >11 mmol/L (>200 mg/dl)	Group 1: <7.8 mmol/L (<140 mg/dl); Group 2: 7.8–11 mmol/L (140–200 mg/dl); Group 3: >11 mmol/L (>200 mg/dl)	209 (13.0)	Primary PCI	30-day mortality: HG (61/279), non-HG (55/1,367) 30-day mortality: HG-DM (42/175), HG-nonDM (19/104) 3-year mortality: HG (20/279), non-HG (94/1,367) 3-year mortality: HG-DM (5/175), HG-nonDM (15/104) Cardiogenic shock: HG (42/279), non-HG (25/1,367) Cardiogenic shock: HG-DM (10/104), HG-nonDM (32/175)	- Non-DM: for every 1 mmol/L blood glucose increase, HR 1.14, (95%CI 1.09–1.19), *p* < 0.01 - DM: for every 1 mmol/L blood glucose increase, HR 1.12 (95%CI 1.05–1.19), *p* < 0.01 - Total cohort: HR 1.13 (95% CI 1.09–1.17), *p* < 0.01
23	Ishihara et al. 2009 (34)	Cohort retrospective	Patients admitted within 48 h of AMI onset	2001–2003 During hospitalization	3,274/3,750	2,690 (71.73) Age 67.69 ± 12.37 years of total sample 3,750	Admission blood glucose ≥ 198 mg/dl (11 mmol/L)	≥198 mg/dl (11 mmol/L)	1,190 (31.74)	Primary coronary angioplasty and thrombolysis	30-days mortality HG (98/1,018), nonHG (107/2,256)	In-hospital mortality Hyperglycaemia adjusted: nondiabetic OR 5.97 (3.57–9.98) *p* < 0.001; diabetic OR 3.51 (1.50–8.24) *p* = 0.004
24	Kalinczuk et al. 2018 (35)	Cohort retrospective	Patients treated for STEMI with pPCI and plasma glycaemia measured from a blood sample taken at the time of introducer sheath insertion (just before the pPCI procedure)	10 months, year not stated (180 days)	323	234 (72.2) Age 60.4 ± 11.5 years	Admission glycaemia > 11.1 mmol/L (or > 200 mg/dl), and either previously diagnosed diabetic subjects or had acute glycaemia >11.1 mmol/L (or > 200 mg/dl)	≥157 mg/dl	55 (17.0)	Primary PCI	6-month mortality HG (15/144), nonHG (7/179)	OR admission blood glucose >= 157 mg/dl = 2.41 (1.36–4.26), *p* = 0.002 (univariate)
25#	Khalfallah et al. 2020 (7)	Cohort prospective	Patients with STEMI who were admitted to cardiovascular department for PPCI without known diabetes history or high HbA1c level.	1January 2017 to 31 December 2018 (3 months)	660	368 (55.8) Age 55.1 ± 9.4 years	Stress hyperglycemia was defined as plasma glucose levels > 140 mg/dl at random (any given time) in hospitalized patients who were not known to have DM	≥140 mg/dl	0 (0)	Primary PCI	3-month mortality HG (9/111), nonHG (19/549) Cardiogenic shock HG (16/111), nonHG (28/549) Cardiac arrest HG (4/111), nonHG (15/549) Heart failure HG (15/111), nonHG (47/549) Cerebral stroke HG (3/111), nonHG (5/549) Reinfarction HG (4/111), nonHG (14/549)	Stress hyperglycemia to mortality OR 2.243 (95%CI 0.947–5.313), *p* = 0.066
26	Kirmani et al. 2022 (36)	Cohort prospective	Patients diagnosed with STEMI undergoing primary PCI	Between March and October 2020 During hospitalization	190	114 (60%) 56.42 ± 11.74	Admission blood glucose > 200 mg/dl	>200 mg/dl	108 (56.8%)	Primary PCI	Heart failure HG (12/60), nonHG (20/130) Recurrent MI HG (2/60), nonHG (0/130) Stroke HG (1/60), nonHG (0/130) Cardiogenic shock HG (2/60), nonHG (0/130) Inhospital mortality HG (2/60), nonHG (0/130) Composite MACCE HG (8/60), nonHG (30/130)	N/A (only available for composite MACCE)
27	Kosuge et al. 2009 (37)	Cohort retrospective	Patients admitted within 48 h of AMI onset	2001–2003 During hospitalization	2,633	1941 (73.72) Age 67 ± 11.75 years	Admission plasma glucose ≥ 188 mg/dl (10.4 mmol/L)	Admission plasma glucose ≥ 188 mg/dl	843 (32.02)	Primary or rescue PCI	In-hospital all cause mortality HG (82/876), non-HG (66/1,757) Reinfarction HG (16/876), non-HG (36/1,757) Heart failure HG (38/876), non-HG (34/1,757) Stroke HG (16/876), non-HG (14/1,757)	All cause in-hospital mortality OR 1.32, 95% CI 1.04 to 2.16, *p* = 0.040
28#	Kruk et al. 2010 (38)	Cohort prospective	STEMI patients with <12 h from symptom to admission	2001–2004 (During hospitalisation)	1,880	1,355 (72.1) Age 60.1 ± 11.8 years	Hyperglycemia was admission serum glucose >11.1 mmol/L (>200 mg/dl)	>200 mg/dl	189 (10.1)	PCI	N/A	Hyperglycemia associated with: Death: HR 2.67 (95% CI 1.56–4.55) *p* < 0.001 Heart Failure: HR 1.12 (95% CI 0.80–1.58) *p* = 0.507 Death or heart failure: HR 1.65 (95% CI 1.20–2.27) *p* = 0.002
29	Kumar et al. 2022 (39)	Cohort prospective	Patients diagnosed with STEMI undergoing primary PCI	Between August 2020 and July 2021 (6 months follow up)	1,102	877 (79.6) 55.66 ± 11.48	Admission blood glucose >200 mg/dl	>200 mg/dl	439 (39.8)	Primary PCI	Composite MACCE HG (91/317), nonHG (119/785)	Composite MACCE 1.9 [1.45–2.5] *p* < 0.001 (univariate); 1.66 [1.25–2.21] *p* < 0.001 (multivariate)
30	Kumar et al. 2023 (40)	Cohort prospective	Patients diagnosed with STEMI undergoing primary PCI	Between January 2022 and June 2022 During hospitalization	4,470	3,517 (78.7%) 55.52 ± 11	Admission blood glucose ≥ 200 mg/dl	≥200 mg/dl	1,586 (35.5)	Primary PCI	Heart failure HG (144/1,759), nonHG (149/2,711) Stroke HG (6/1,759), nonHG (8/2,711) Inhospital mortality HG (101/1,759), nonHG (69/2,711)	In-hospital mortality OR 1.81 (1.28–2.55), *p* < 0.001
31	Lavi et al. 2008 (41)	Cohort prospective	Ongoing registry of all consecutive ST-segment elevation acute myocardial infarction patients who underwent primary PCI, without DM	between January 1996 and June 2004 (1 year)	343	292 (85.1) Age 56.7 ± 12.0 years	Hyperglycemia was defined as the presence of fasting plasma glucose >126 mg/dl (>7.0 mmol/L; diabetic range glucose level) in non-diabetic patients, during the first day of hospitalization, after overnight fasting	Fasting blood glucose >126 mg/dl	0 (0)	Primary PCI	In-hospital mortality HG (10/119), nonHG (3/224) Reinfarction HG (3/119), nonHG (3/224) Heart failure HG (29/119), nonHG (27/224) 1-year mortality HG (12/119), nonHG (3/224)	In-hospital heart failure HG vs nonHG in non diabetics: OR: 3.2, CI: 1.5–6.7; *p* = 0.002 1-year mortality HG vs nonHG in non diabetics: OR: 0.08, CI: 0.03–0.3; *p* = 0.001
32	Lazzeri et al. 2011 (42)	Cohort prospective	STEMI patients within 12 h from symptoms onset, with and without previously known diabetes, admitted to ICCU after primary PCI	30 June 2008 to 30 June 2010 (During hospitalisation)	611	451 (73.8) Age 67 (58–76) years	Plasma glucose >180 mg/dl	In-hospital peak glycaemia > 180 mg/dl	115 (18.8)	Primary PCI	In-hospital mortality HG (31/206), nonHG (4/405)	OR peak glycaemia 3.214 (1.368–7.554), *p* = 0.007
33	Lazzeri et al. 2013 (43)	Cohort prospective	Non-diabetic patients with STEMI undergone mechanical revascularization	30 June 2008 to 30 June 2009 (During hospitalisation)	356	263 (74.0) Age 65 (54–80) years	Fasting blood glucose > 140 mg/dl	>140 mg/dl	0 (0)	Primary PCI	In-hospital mortality HG (11/130), non-HG (1/226) In-hospital MACE HG (34/109), non-HG (25/125)	HG and ICCU mortality: OR 7.39 (95%CI 2.70–20.20) HG and ICCU complications: OR 1.79 (95%CI 1.09–2.93)
34	Lazzeri et al. 2013 (43)	Cohort prospective	Patients with first episode of STEMI ≤12 h from symptom onset) admitted to ICCU, after primary PCI, without previously known DM	1 January 2004 to 31 December 2011 (1 year)	1,205	686 (73.9) Age 65 (56–76) years	HbA1c > 6.5%	HbA1c > 6.5%	0 (0)	Primary PCI	In-hospital mortality HG (10/78), nonHG (30/851) 1-year post discharge mortality HG (5/58), nonHG (35/646)	All cause-mortality HR 1.16 (0.92–1.46), *p* = 0.218
35	Liu et al. 2012 (44)	Cohort prospective	Patients with a clinical diagnosis of STEMI admitted to 247 hospitals in China within 12 h of the onset of symptom and without diabetes history	June 2001 to July 2004 (30 days)	4,793	3,427 (71.5) Age 62.6 ± 11.9 years	Mean blood glucose and admission blood glucose ≥173 mg/dl HbA1c ≥ 6.5%	Based on admission blood glucose quintiles, HbA1c ≥ 6.5%; Mean blood glucose and admission blood glucose ≥173 mg/dl	0 (0)	Both fibrinolysis and PCI	Mean blood glucose: Mortality at 7 days, HG (147/954), nonHG (218/3,837) Mortality at 30 days, HG (176/954), nonHG (310/3,837) Admission blood glucose: Mortality at 7 days, HG (132/954), nonHG (241/3,837) Mortality at 30 days, HG (156/954), nonHG (330/3,837)	Mortality 7 days Mean blood glucose: 1.14 (1.10–1.18), *p* < 0.05 Admission blood glucose: 1.07 (1.04–1.09), *p* < 0.05 Mortality 30 days Mean blood glucose 1.12 (1.08–1.15), *p* < 0.05 Admission blood glucose 1.05 (1.03–1.08), *p* < 0.05
36^#^	Luo et al. 2019 (45)	Cohort retrospective	Consecutive patients with STEMI admitted to Zhongda Hospital aged 18–80 years old which treated with PCI	January 2012 to March 2018 (1 year)	1,092	874 (80.0) Age 61.9 ± 12.0 years	TyG index ≥ 9.608; or Fasting blood glucose (FBG) level > 7.0 mmol/L (blood sample was taken after overnight fast ≥ 12 h)	FBG ≥126 mg/dl, TyG index ≥ 9.608	270 (24.7)	Primary or rescue PCI	30-day mortality: TyG high (12/273), TyG low (12/819) 6-month mortality: TyG high (24/273), TyG low (20/819) 1-year mortality: TyG high (29/273), TyG low (27/819)	N/A for mortality, only MACCE without specifying type of MACCE
37	Marenzi et al. 2010 (46)	Cohort prospective	STEMI patients undergoing primary PCI	2003–2008 During hospitalization	780	633 (81.16) Age 62 ± 12 years	Admission glucose levels >198 mg/dl	>198 mg/dl	109 (13.98)	Primary or rescue PCI	In-hospital mortality HG (18/148), non-HG (19/632) Cardiogenic shock HG (32/148), non-HG (44/632)	In-hospital mortality = RR 4.05 (2.18–7.52), *p* < 0.001
38	Mladenović et al. 2010 (47)	Cohort prospective	patients with STEMI	January, 1 2007 to June 30, 2007 1 year	115	81 (69.6) Age 64.3 ± 10.7 years	Admission blood glucose > 180 mg/dl	>180 mg/dl	11,529 (25.2)	N/A	30-days mortality HG (7/16), nonHG (9/70) 1-year mortality HG (9/16), nonHG (19/70)	High APG level is common in patients with STEMI and associated with high risk of mortality and morbidity. Nondiabetic patients with high APG have higher risk of mortality than patients with a known history of diabetes
39	Moura et al. 2015 (48)	Cohort prospective	STEMI patients	2006–2013 2 years	326	155 (47.5) Age 60.9 ± 11.0 years	Hyperglycaemic if HbA1c ≥ 5.8%	HbA1c ≥ 5.8%	0 (0)	Fibrinolysis or PCI	30-days mortality HG (10/152), non-HG (12/174)	Elevated HbA1c is associated with adverse long-term prognosis in non-diabetic STEMI patients treated mainly with thrombolytics
40	Orellana-Barrios et al. 2019 (49)	Cohort retrospective	Patients with STEMI who had recent HbA1c measurements (on admission and up to 6 months prior to admission) aged 18 to 89 years	1 January 2010 to 1 May 2015 (12 months)	676 (available in-hospital mortality data based on admission glucose is 230)	500 (74.0) Age 59.9 ± 13.4 years	Acute hyperglycaemia: admission glucose ≥ 200 mg/dl Chronic hyperglycaemia: AG ≥ 200 mg/dl. A significant acute glucose delta was an admission glucose delta ≥140 mg/dl	Admission glucose ≥200 mg/dl and admission glucose delta ≥ 140 mg/dl	189 (27.9)—not known in 230 patients subgroups	Both fibrinolysis and PCI	In-hospital mortality HG (6/77), nonHG (6/153)	Non-adjusted OR 4.14 (0.71–24.47), *p* = 0.176
41	Porter et al. 2008 (50)	Cohort prospective	Patients without previously known diabetes mellitus who were treated with PPCI for ST-elevation AMI	Between January 2001 and June 2006 (6 months)	570	487 (85.4) Age 60.1 ± 12.9 years	Fasting blood glucose ≥126 mg/dl (after >8 h of fasting)	≥ 126 mg/dl	0 (0)	Primary PCI	30-day mortality HG (2/39), nonHG (13/531) 30-day stent thrombosis HG (2/39), nonHG (17/531) 30-day recurrent MI HG (1/39), nonHG (19/531) 6-month mortality HG (4/39), nonHG (23/531) 6-month recurrent MI HG (2/39), nonHG (27/531)	30-day mortality Significantly impaired FG (groups 3–4, FG ≥110 mg/dl) OR 1.70 (95%CI 1.03–2.70), *P* = 0.04
42	Pres et al. 2010 (51)	Cohort retrospective	Patients with STEMI complicated with cardiogenic shock who were treated with PCI, with time from the symptom onset to PCI ≤18 h	From 1998 to 2006 (5 years)	258	169 (65.5) Age 61.9 ± 10.8 years	Admission glucose level ≥140 mg/dl	≥140 mg/dl	79 (30.6)	PCI	In-hospital mortality HG (76/183), non-HG (21/75) Emergency CABG HG (6/183), non-HG (2/75) 1-year mortality HG (94/183), non-HG (26/75) 5-year mortality HG (65.8%), non-HG (43.3) Data for 106 patients	Admission glucose was an independent prognostic factor of in-hospital mortality OR 1.08 (1.02–1.14), *p* = 0.0044 1-year mortality HR 1.04 (1.01–1.06), *p* = 0.005 5-year mortality HR 1.03 (1.01–1.05), *p* = 0.045
43	Pusuroglu et al. 2014 (52)	Cohort prospective	Patients admitted to a large-volume tertiary training and research hospital with a diagnosis of STEMI within 12 h of symptom onset or for up to 18 h if there was evidence of continuing ischaemia or haemodynamic instability aged 18 and 80 years old, who underwent primary PCI	September 2010 and July 2012 (1 year)	443	362 (81.7) Age 54.7 ± 12.0 years	Diabetes was defined as HbA1c ≥ 6.5%	HbA1c ≥ 6.5%	82 (18.5)	Primary PCI	In-hospital CV mortality HG (5/129), nonHG (9/314) In-hospital recurrent MI HG (10/129), nonHG (12/314) In-hospital stroke HG (0/129), nonHG (2/314) In-hospital HF HG (11/129), nonHG (20/314) In-hospital cardiogenic shock HG (5/129), nonHG (12/314) In-hospital stent thrombosis HG (9/129), nonHG (13/314) 30-day CV mortality HG (8/129), nonHG (13/314) 30-day nonfatal recurrent MI HG (10/129), nonHG (15/314) 30-day stroke HG (0/129), nonHG (2/314) 30-day advanced HF HG (28/129), nonHG (57/314) 1-year CV mortality HG (10/129), nonHG (17/314) 1-year nonfatal recurrent MI HG (17/129), nonHG (18/314) 1-year stroke HG (4/129), nonHG (5/314) 1-year advanced HF HG (22/129), nonHG (50/314)	1-year cardiovascular mortality HbA1c OR 1.132 (95%CI 0.913–1.402), *p* = 0.258
44	Qanitha et al. 2018 (53)	Cohort prospective	Patients with ACS and stable CAD	Cohort 1: From February 2013 to December 2014; Cohort 2: 2018–2020 (6 months)	576	455 (79.0) Age 56.8 ± 11.1 years	Admission glucose ≥200 mg/dl	≥200 mg/dl	154 (26.7)	Both fibrinolysis and PCI	In-hospital mortality: HG (15/124), non-HG (47/452) 30-day mortality: HG (19/124), non-HG (50/452) 6-month mortality: HG (28/124), non-HG (84/452) 6-month HF hospitalization: HG (15/124), non-HG (44/452) 6-month Reinfarction: HG (10/124), non-HG (20/452) LVEF <35%: HG (20/105), non-HG (71/386)	Hyperglycemia is an independent predictor of all-cause mortality adjusted OR 1.55 (95%CI 1.12–2.14), *P* = 0.008
45	Qiao-Qin et al. 2020 (54)	Cohort retrospective	ST-segment elevation acute myocardial infarction (STEMI) patients undergoing emergency coronary angiography	2016–2018 During hospitalization	958	744 (77.67) Age 62.3 ± 13.3 years	Hyperglycemia if fasting blood glucose (FBG) > 110 mg/dl or admission blood glucose (ABG) > 200 mg/dl	FBG > 110 mg/dl or ABG > 200 mg/dl	262 (27.35)	N/A	Admission blood glucose (ABG) In-hospital mortality HG (56/265), non-HG (24/693) Recurrent MI HG (6/265), non-HG (4/693) Stroke HG (1/265), non-HG (0/693) Cardiogenic shock HG (57/265), non-HG (49/693) Fasting blood glucose (FBG) In-hospital mortality HG (72/589), non-HG (5/369) Recurrent MI HG (7/589), non-HG (3/369) Stroke HG (1/589), non-HG (0/369) Cardiogenic shock HG (89/589), non-HG (17/369)	In-hospital adverse events: ABG: OR 1.205 (1.04–1.63), *p* = 0.048 FBG: OR 1.740 (1.12–1.98), *p* = 0.043
46	Qin et al. 2022 (55)	Cohort retrospective	STEMI patients with percutaneous coronary intervention (PCI) conducted immediately upon hospital admission	Between January 2014 and October 2019 (follow-up 1 year)	1,098	826 (75.2) Age 63.9 ± 13.1 years	Admission hyperglycemia: glucose level ≥ 180 mg/dl (≥10 mmol/L) was selected as the cutoff value in accordance with the guidelines provided by the American Diabetes Association (ADA).	Diabetic: admission glucose ≥ 180 mg/dl vs. < 180 mg/dl Non-diabetic: admission glucose ≥ 200 mg/dl vs. < 200 mg/dl	272 (24.8)	Primary or rescue PCI	1-year total MACCE HG (68/242), non-HG (154/856) 1-year cardiac death HG (16/242), non-HG (20/856) 1-year non-fatal MI HG (21/242), non-HG (42/856) 1-year revascularization HG (28/242), non-HG (59 /856) 1-year HF or angina hospitalisation HG (38/242), non-HG (98/856) 1-year ischemic stroke HG (2/242), non-HG (8/856) 1-year cardiogenic shock HG (45/242), non-HG (56/856) Length of hospitalisation (days) HG (9.7 ± 4.6), non-HG (9.0 ± 3.9) DM HG vs. Non-DM HG 1-year total MACCE: DM HG (36/158), non-DM HG (32/84) 1-year cardiac death: DM HG (6/158), non-DM HG (10/84) 1-year non-fatal MI: DM HG (12/158), non-DM HG (9/84) 1-year revascularization DM HG (19/158), non-DM HG (9/84) 1-year HF or angina hospitalisation DM HG (22/158), non-DM HG (16/84) 1-year ischemic stroke DM HG (2/158), non-DM HG (0/84) Length of hospitalisation (days) DM HG [9 (6–11.3)], non-DM HG [6.5 (5–9)]	Admission hyperglycemia associated with 1-year MACCE with OR 1.8 (95%CI 1.3–2.1), *p* = 0.001
47	Rasoul et al. 2007 (56)	Cohort prospective	First time STEMI patients, none with previously documented diabetes mellitus	April 2002 to February 2004 (Mean 1.6 ± 0.6 years)	504	362 (71.8) Age 63.2 ± 12.8 years	Hyperglycaemia was defined as admission glucose ≥11.1 mmol/L based on nonfasting cut-off values for hyperglycaemia from the guidelines of the American Diabetic Association. An HbA1c ≥ 6.0% was considered an elevated HbA1c	Admission blood glucose ≥200 mg/dl; Elevated HbA1c ≥ 6%	0 (0)	Primary or rescue PCI	Admission blood glucose 30-day mortality HG (15/81), nonHG (17/415) > 6 months mortality HG (3/66), nonHG (17/398) HbA1c 30-day mortality HG (10/86), nonHG (22/408) > 6 months mortality HG (5/76), nonHG (15/388)	30-day mortality elevated admission blood glucose—OR 4.91, (2.03 to 11.9), *p* < 0.001 Elevated HbA1c—OR 1.33 (0.48 to 3.71), *p* = 0.58
48	Rousan et al. 2014 (57)	Cohort retrospective	Patients with ST-segment elevation myocardial infarction (STEMI) enrolled at 609 hospitals across the United States	1 January 2007 to 31 March 2011 (in hospital)	93,569	65,974 (70.5) Age 59.7 (51–72) years	Initial HbA1C level >6.5% on presentation	HbA1C level > 6.5%	2,157 (23.0)	Fibrinolysis, PCI, and OMT	DM vs nonDM In-hospital mortality DM (1,076/21,507), nonDM (2,306/72,062) Cardiogenic shock DM (1,312/21,507), nonDM (3,459/72,062) HbA1c > 6.5% In-hospital mortality HG (65/2,591), nonHG (360/16,325) Cardiogenic shock HG (125/2,591), nonHG (915/16,325)	In-hospital mortality in DM patients—OR 1.17 (1.07 to 1.27), *p* < 0.05
49	Shahid et al. 2020 (58)	Cohort prospective	STEMI patients	2018–2019 During hospitalization	256	196 (76.5) Age 55 ± 11 years	Admission blood glucose levels >140 mg/dl	>140 mg/dl	92 (35.93)	Fibrinolysis or PCI	In-hospital mortality HG (12/96), non-HG (6/160)	Hyperglycemia is independently associated with cardiac death in STEMI patients, and the predictive value of hyperglycemia is higher in T2DM patients compared to patients without T2DM
50	Shitole et al. 2020 (59)	Cohort prospective	STEMI patients in which some patients with the initial glucose value ≥180 mg/dl with a glucose. Cardiogenic shock on presentation was excluded	May 2008 to December 2014 (Median of 4.6 years)	1,067	721 (67.6) Age 59 (50–69) years	Admission blood glucose ≥180 mg/dl	180 mg/dl	348 (32.6), newly diagnosed diabetes among nondiabetic paatients—43 (5.9)	Primary or rescue PCI	In-hospital mortality HG (15/354), nonHG (13/713) 30-day mortality HG (18/354), nonHG (15/713)	Patients with pronounced hyperglycaemia had correspondingly higher adjusted risks of death and combined death or CVD readmission at 1 year and through the duration of follow- up, as compared to patients with normal glucose regulation
51	Sinnaeve et al. 2009 (60)	Cohort prospective	Adult patients (>18 years old) admitted with a presumptive diagnosis of an ACS (STEMI vs. NSTEMI vs. UA)	1 April 1999 to 31 December 2005 (6 months)	5,351	3,563 (66.6) Age 66 (56–76) years	Fasting and admission glucose ≥126 mg/dl, fasting blood glucose was documented at any time during the hospitalization	Normal fasting glucose levels (<100 mg/dl), impaired fasting glucose levels (100–125 mg/dl), Hyperglycaemia (126–199, 200–299, and ≥300 mg/dl)	1,498 (28.0)	Both fibrinolysis and PCI	Admission blood glucose In-hospital mortality HG (291/3,751), non-HG (392/9,775) 6-month mortality HG (221/3,751), non-HG (485/9,775) Fasting blood glucose In-hospital mortality HG (394/3,751), non-HG (294/9,775) 6-month mortality HG (593/3,751), non-HG (328/9,775)	Admission BG: 126–199 mg/dl- OR 1.14 (0.69–188) 200–299 mg/dl- OR 0.77 (0.40–1.45) ≥300 mg/dl- OR 0.83 (0.39–1.73) Fasting BG: 126–199 mg/dl- OR 2.30 (1.39–3.81) 200–299 mg/dl -OR 1.04 (0.37–2.95) ≥300 mg/dl- OR 6.30 (2.11–18.70)
52	Squire et al. 2010 (61)	Cohort retrospective	Patients with STEMI	From 1 April 1993 to 31 December 2005 (1 year)	4,702	3,198 (68.0) Age 66.7 ± 12.7 years	Admission glucose ≥11 mmol/L	Quartile 1 (< 7 mmol/L) Quartile 2 (7–8.2 mmol/L) Quartile 3 (8.3–10.9 mmol/L) Quartile 4 (>11 mmol/L)	749 (15.9)	N/A	30-day mortality: HG (329/1,059), non-HG (369/2,959) 1-year mortality: HG (405/1,021), non-HG (530/2,818)	For patients with glucose ≥11 mmol/L), 30-day mortality was over 3-fold higher than those with glucose <7 mmol/L (31% vs. 9.0%). 30-day mortality: OR 4.54 (95% CI 3.50, 5.88). 1-year mortality: OR 4.04 (95% CI 3.21, 5.07)
53	Stalikas et al. 2022 (62)	Cohort prospective	Patients with STEMI undergoing coronary angiography in a tertiary academic hospital	From July 2019 to May 2021 (Median follow-up 1.7 years)	309	249 (80.3) 60.4 ± 12.0	Fasting blood glucose (fasted at least 8 h) > 140 mg/dl	> 140 mg/dl	57 (18.5)	Primary PCI	1 year Heart failure (cardiac hospitalization) HG (4/121), nonHG (7/188) Stroke HG (2/121), nonHG (0/188) Mortality HG (31/121), nonHG (22/188)	N/A (only available for composite MACCE)
54	Straumann et al. 2005 (63)	Cohort prospective	all patients undergoing PCI during the first 24 h of an acute MI	N/A 5 years	978	785 (80.27) Age 60.3 ± 12.0 years	Admission blood glucose > 198 mg/dl (> 11 mmol/L)	> 198 mg/dl	172 (17.59)	Primary or rescue PCI	30-days mortality HG (52/308), non-HG (26/670) 3-years mortality HG (82/308), non-HG (57/670)	30-days mortality RR 3.92 (1.17–13.17) *p* = 0.027 3 years mortality RR 1.76 (1.01–3.08) *p* = 0.047
55	Takada et al. 2012 (64)	Cohort prospective	Patients with ACS	January 2004 to April 2007 (During hospitalization)	212	152 (71.7) Age 59.2 ± 12.8 years	Admission blood glucose > 200 mg/dl	>200 mg/dl	55 (25.9)	Both fibrinolysis and PCI	In-hospital mortality HG (6/59), nonHG (2/153)	not available because insignificant
56	Terlecki et al. 2013 (4)	Cohort prospective	Patients admitted with STEMI who treated with an early invasive management strategy	2004–2007 (During hospitalization)	246	166 (67.5) Age 64.5 (55–72) years	Acute hyperglyemia was defined as glucose on admission ≥ 7.8 mmol/L (≥140 mg/dl)	≥140 mg/dl	75 (30.5)	Primary or rescue PCI	In-hospital mortality HG (16/136), nonHG (2/110) Cardiogenic shock HG (14/136), nonHG (1/110) HF hospitalization HG (60/136), nonHG (22/110) Stroke/TIA HG (1/136), nonHG (0/110) Repeat PCI HG (7/136), nonHG (0/110) Emergency CABG HG (6/136), nonHG (4/110)	In-hospital risk of death and/or cardiogenic shock Acute hyperglycaemia and low leukocyte count OR 4.2 (95% CI: 0.42–41.66) *p* = 0.22 Acute hyperglycaemia and elevated leukocyte count OR 17.6 (95% CI: 1.88–165.26), *p* = 0.0122
57	Tian et al. 2012 (65)	Cohort prospective	STEMI patients treated with primary PCI at 26 hospitals in China who presented with typical complaints of chest pain within 12 h from the symptom onset	June 2001 to July 2004 (30 days)	608	481 (79.1) Age 61.4 ± 11.4 years	HbA1c ≥ 6.5%	HbA1c ≥ 6.5%	98 (16.1)	Primary PCI	7-day mortality HG (0/164), nonHG (9/444) 30-day mortality HG (2/164), nonHG (14/435)	HR HbA1c 0.431 (0.175–1.061), *p* = 0.067
58	Timmer et al. 2011 (66)	Cohort prospective	STEMI patients without known DM and treated with primary PCI	January 2004 to January 2009 (mean 3.3 ± 1.5 years)	4,176	3,092 (74.0) Age 62.5 ± 13.0 years	HbA1c ≥ 5.55% Admission glucose ≥9.6 mmol/L (≥172 mg/dl)	HbA1c quartiles: ≤5.35%, 5.36–5.54%, 5.55–5.80%, ≥5.81% Admission glucose quartiles: ≤124 mg/dl, 125–144 mg/dl, 145–171 mg/dl, ≥172 mg/dl	0 (0)	Primary PCI	HbA1c: 30-day mortality HG (56/2,066), non-HG (45/2,110) 1-year mortality HG (121/2,066), non-HG (75/2,110) Admission Glucose: 30-day mortality HG (506/1,032), non-HG (498/3,100) 1-year mortality HG (83/1,032), non-HG (112/3,100)	HbA1c for long-term mortality per IQR: HR 1.2 (1.0–1.3), *p* < 0.010
59#	Vis et al. 2010 (67)	Cohort prospective	STEMI patients presented with cardiogenic shock on admission and underwent PCI	From January 1997 to March 2005 (1 year)	292	120 (66.0) Age 63.0 ± 13.0 years	N/A	N/A	0 (0)	Primary or rescue PCI	N/A	Admission glucose associated with 1-year mortality with OR 1.11 (95% CI 1.02–1.21, *P* = 0.013)
60	Wang et al. 2021 (68)	Cohort retrospective	STEMI patients who underwent primary PCI	2003–2015 30 days	623	514 (82.5) Age 61.3 ± 12.9 years	Fasting blood glucose (FBG) > 6.1 mmol/L or 110 mg/dl	FBG > 110 mg/dl	0 (0)	Primary or rescue PCI	30-days all cause mortality HG (9/161), non-HG (5/462) Heart failure HG (44/161), non-HG (46/462) Recurrent MI HG (1/161), non-HG (7/462) Cardiac shock HG (15/161), non-HG (19/462)	HR 1.273 (1.168–1.388), *p* < 0.001
61#	Weston et al. 2007 (69)	Cohort retrospective	Patients with a final diagnosis of troponin- positive acute coronary syndrome who were not previously known to have diabetes mellitus	2003–2006 30 days	1,267/2,777	1,506 (57.12) Age 74.83 (63–82) years of 2,637 sample	Admission blood glucose ≥198 mg/dl (≥ 11 mmol/L)	Admission blood glucose ≥ 198 mg/dl	0 (0)	Thrombolytic and primary angioplasty	7-days mortality HG (231/1,264) 30-days mortality HG (273/1,264)	Adjusted RR 7-days mortality = 1.62 (1.18–2.22), *p* = 0.003 30-days mortality = 1.58 (1.19–2.10), *p* = 0.002
62	Worthley et al. 2007 (70)	Cohort prospective	Patients STEMI and treated with primary angioplasty	Between January 2002 and December 2004 (30 days)	980	720 (73.5) Age 62.0 years	Admission glucose level >7.8 mmol/L (>140 mg/dl)	Group 1 (≤6.6 mmol/L or ≤119 mg/dl), Group 2 (6.7–7.8 mmol/L or 120–140 mg/dl), Group 3 (7.9–10.0 mmol/L or 141–180 mg/dl), Group 4 (≥10.1 mmol/L or ≥181 mg/dl).	157 (16.0)	Primary angioplasty	In-hospital mortality HG (30/478), non-HG (7/502) In-hospital mortality HG-DM (23/237), HG non-DM (14/743) Cardiogenic shock HG (48/478), non-HG (12/502)	The risk-adjusted OR 1.10 (95% CI, 1.02- 1.19) for each millimolar increase in blood glucose level value.
63	Xu-hua et al. 2006 (71)	Cohort prospective	STEMI patients treated with primary PCI within 12 h of the symptom onset	From October 2001 to January 2005 (30 days)	308	216 (70.1) Age 61.0 ± 11.0 years	Admission glucose level ≥11.0 mmol/L (≥200 mg/dl)	Admission glucose: Group 1 (<7.8 mmol/L); Group 2 (7.8–11.0 mmol/L); and Group 3 (≥11.0 mmol/L)	N/A	Primary PCI	In-hospital death HG (7/66), non-HG (10/242) 30-day MACCE HG (10/66), non-HG (26/242) LVEF HG [EF 53.8 (10)], non-HG [EF 57.9 (9)]	Elevated admission glucose levels in ST—segment elevation myocardial infarction patients treated with primary PCI are independently associated with impaired microvascular flow
64	Yang et al. 2013 (72)	Cohort prospective	STEMI patients with cardiogenic shock	November 2005 to September 2010 (30 days)	816	513 (62.9) Age 67.3 (56.0–76.0) years	Admission blood glucose ≥11.0 mol/L (≥200 mg/dl)	Group 1: < 7.8 mmol/L (<140 mg/dl); Grup 2: 7.8–10.9 mol/L (140–199 mg/dl) Group 3: 11.0–16.5 mmol/L (200–299 mg/dl) Group 4: ≥ 16.6 mmol/L (≥300 mg/dl)	239 (29.3)	Both fibrinolysis and PCI	30-day mortality HG (174/420), non-HG (87/396) 30-day mortality with DM HG (72/186), non-HG (14/53) 30-day mortality non DM HG (102/234), non-HG (73/343)	30-day mortality nonDM: OR 1.76 (1.06–2.94), *p* 0.026 30-day mortality with DM OR 1.74 (0.59–5.18), *p* 0.507
65	Yanishi et al. 2016 (73)	Cohort prospective	Patients with AMI (STEMI) who received CAG or PCI	2009–2012 During hospitalization	1,060 of total 1,581	1,129 (71.41) Age 70.0 ± 12.6 years	Admission blood glucose > 200 mg/dl	>200 mg/dl	496 (31.38)	CAG or PCI	In-hospital mortality HG (65/302), non-HG (50/758)	In-hospital mortality OR 3.44 (2.18–5.41) *p* = n/a
66	Zhang et al. 2013 (15)	Cohort retrospective	Patients with STEMI without diabetes mellitus consecutively referred to the catheterization laboratory for PPCI in two large hospitals	From January 2007 to October 2011 and from January 2008 to October 2011 (30 days)	853	600 (70.3) Age 62.2 ± 10.6 years	Admission blood glucose ≥180 mg/dl	≥180 mg/dl	0 (0)	Primary PCI	In-hospital mortality HG (9/172), nonHG (16/681) Stent thrombosis in hospital HG (7/172), nonHG (9/681)	in-hospital mortality -OR 1.83 (95%CI 1.52–2.14), *p* = 0.024 Stent thrombosis—OR 1.47 (95%CI 1.18–1.75), *p* = 0.016

^#^ Studies were not included in meta-analysis.

### Participant's characteristics

In total, 164,927 patients were included in the study, with 72.9% being male, and a mean age of 58.5 years. Only a third of the patients had a known history of diabetes mellitus. The majority of the patients received emergency or primary PCI as the treatment for STEMI. The minimum blood glucose level used as a hyperglycemia cutoff was 110 mg/dl.

### Quality assessment

**Figure 2:**
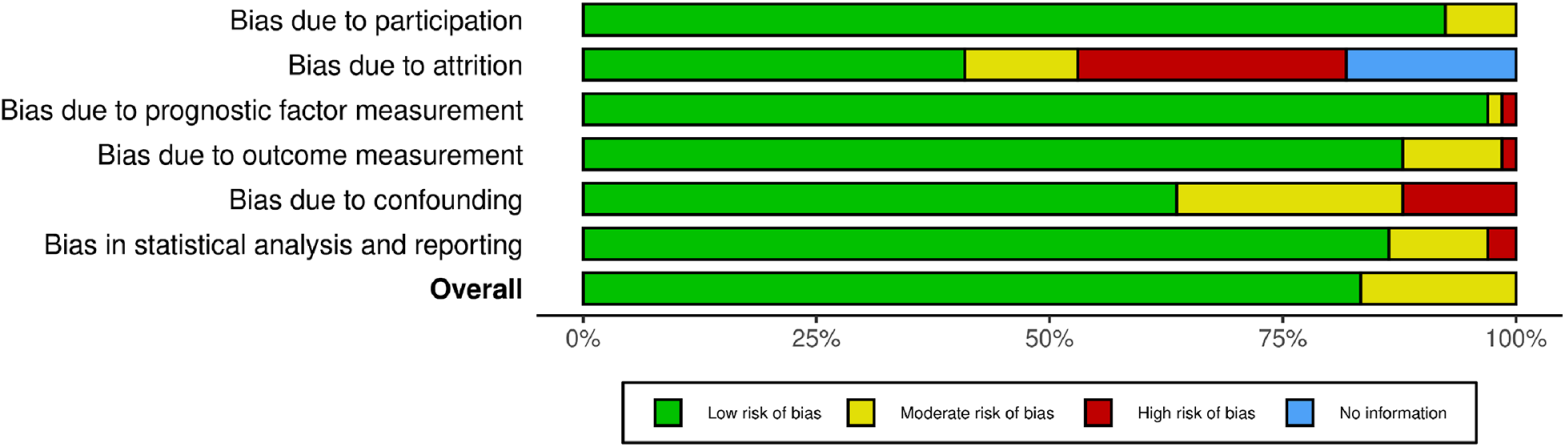
Risk of bias (RoB) assessment of the included studies.

We assessed the quality of the included studies using *the Quality of Prognosis Studies (QUIPS)* tool, designed for the evaluation of risk of bias in observational cohort studies. The summary of the quality assessment is presented in Figure 2 and Supplementary Figure S1, indicating a range of quality from low to moderate risk.

### Hyperglycemia and STEMI outcomes

We analyzed outcomes in subgroups based on glucose parameters: admission blood glucose, HbA1c, and fasting blood glucose. Results were presented in forest plots analyzed using random effects. Pooled risk ratio (RR) and 95% confidence interval (CIs) were calculated for each outcome (Figures 3A–C).

**Figure 3A:**
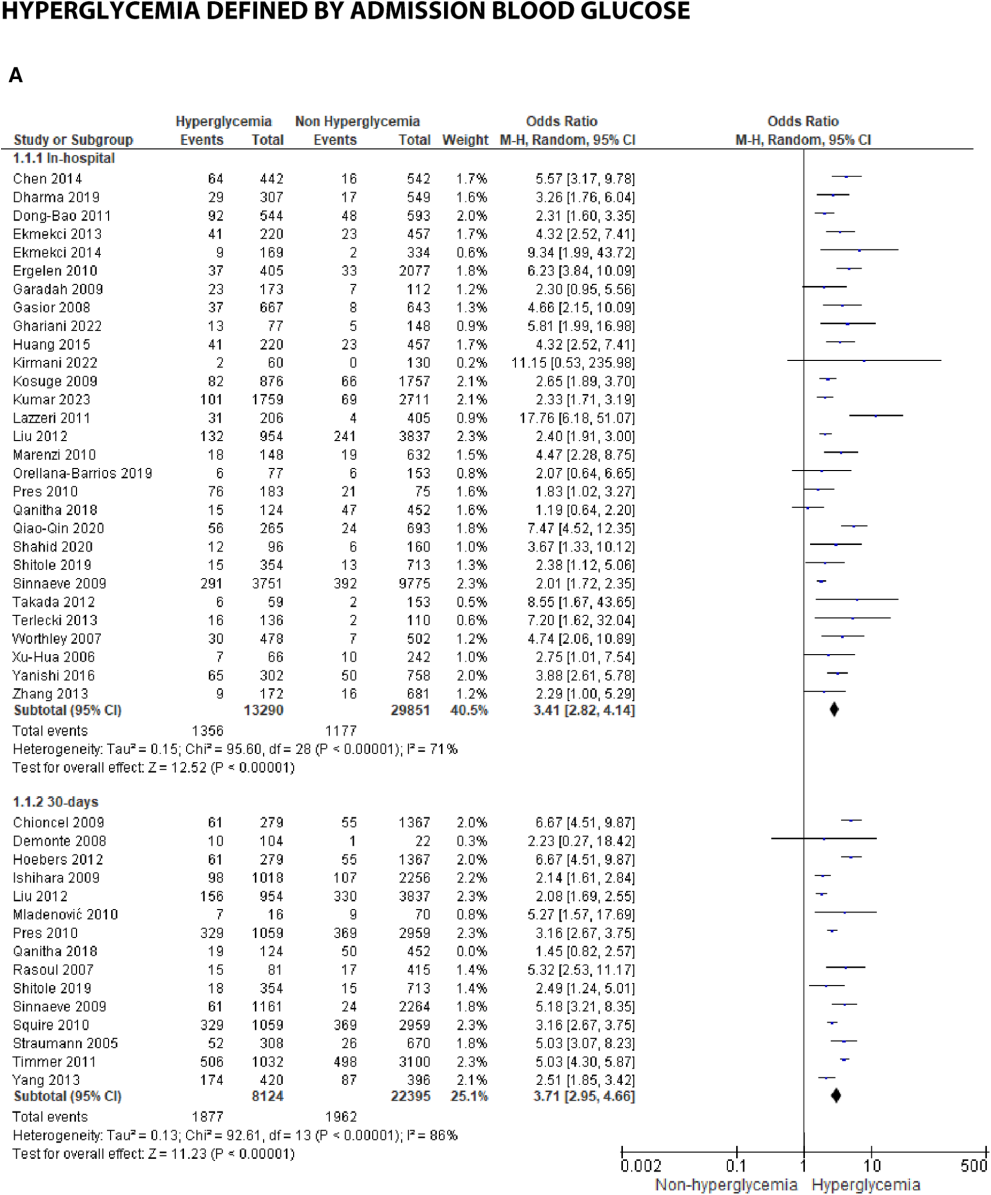
Forest plot of studies using admission blood glucose as a parameter for hyperglycemia with outcomes of (**A**) mortality (in-hospital, 30 days, 6 months, and >6 months), (**B**) reinfarction or recurrent MI, (**C**) heart failure, (**D**) stroke, (**E**) cardiogenic shock, (**F**) repeat PCI or stent thrombosis, (**G**) composite MACCE, and (**H**) emergency CABG.

**Figure:**
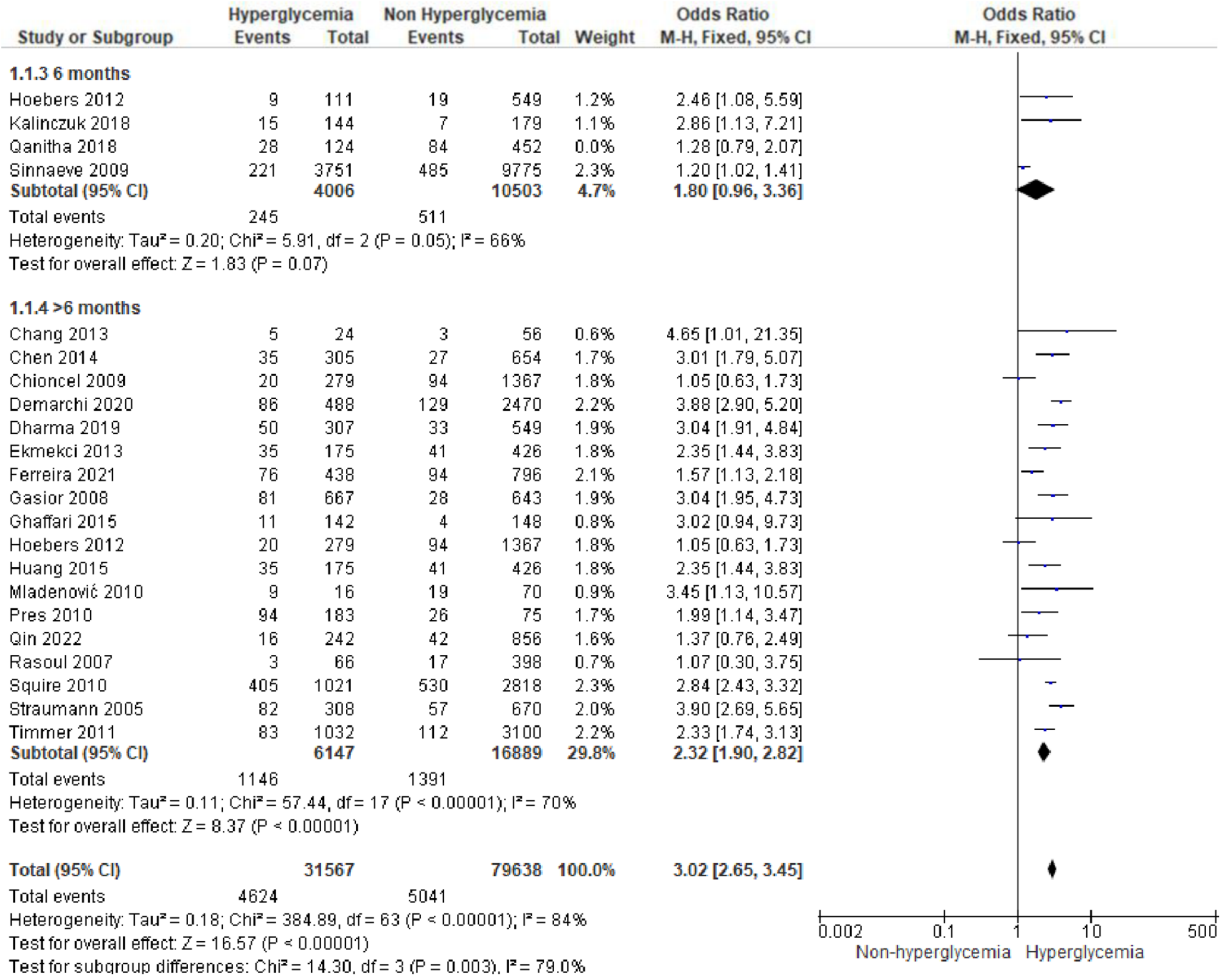


**Figure:**
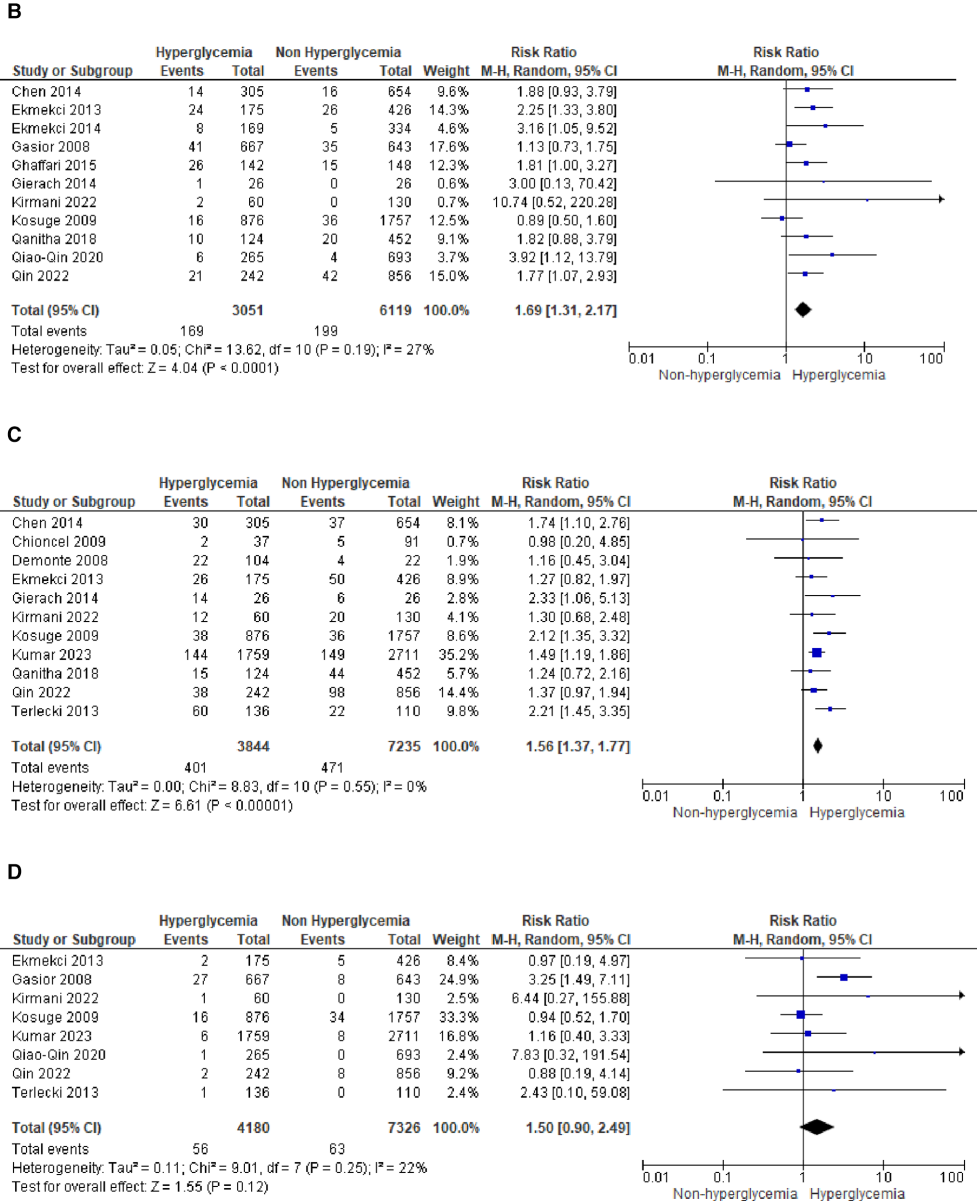


**Figure:**
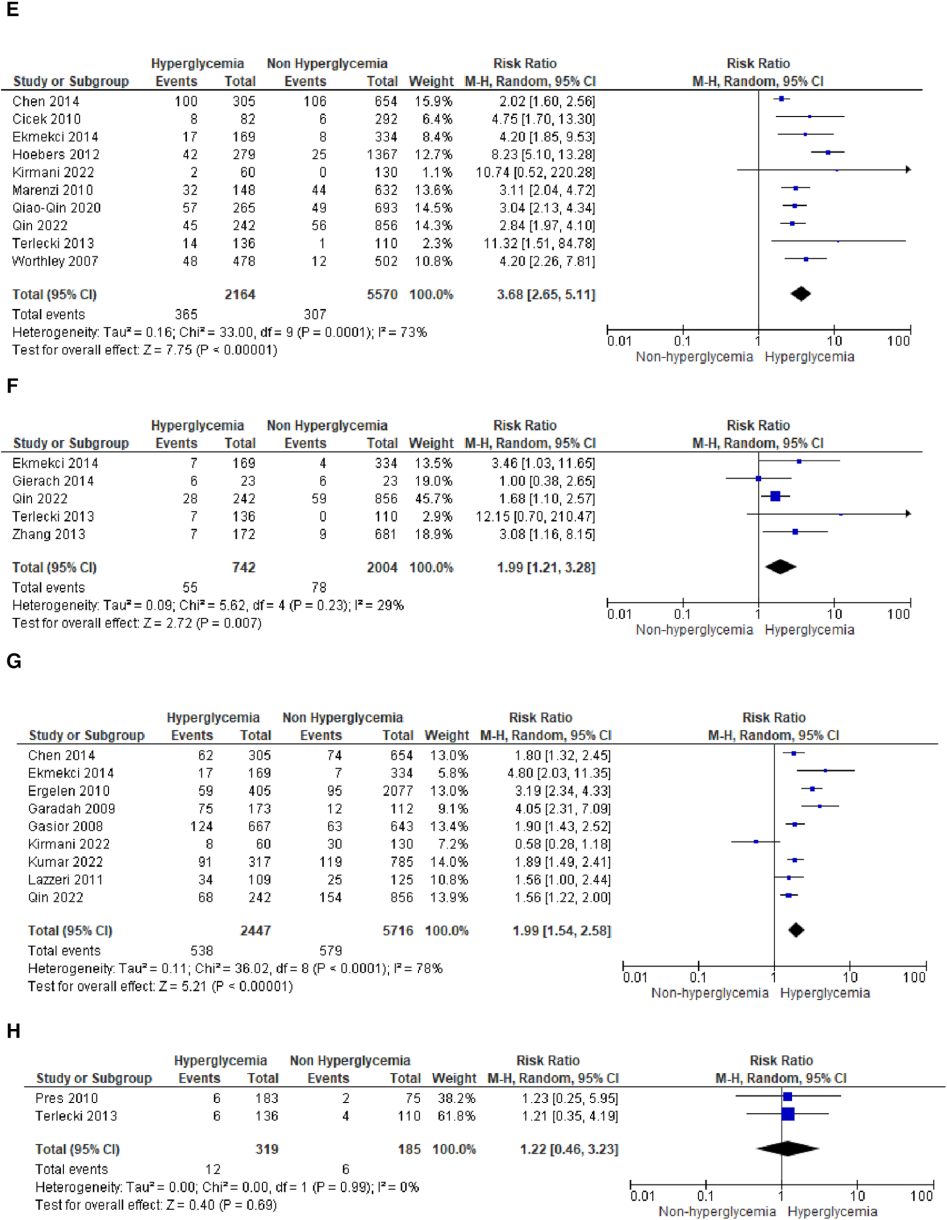


**Figure 3B:**
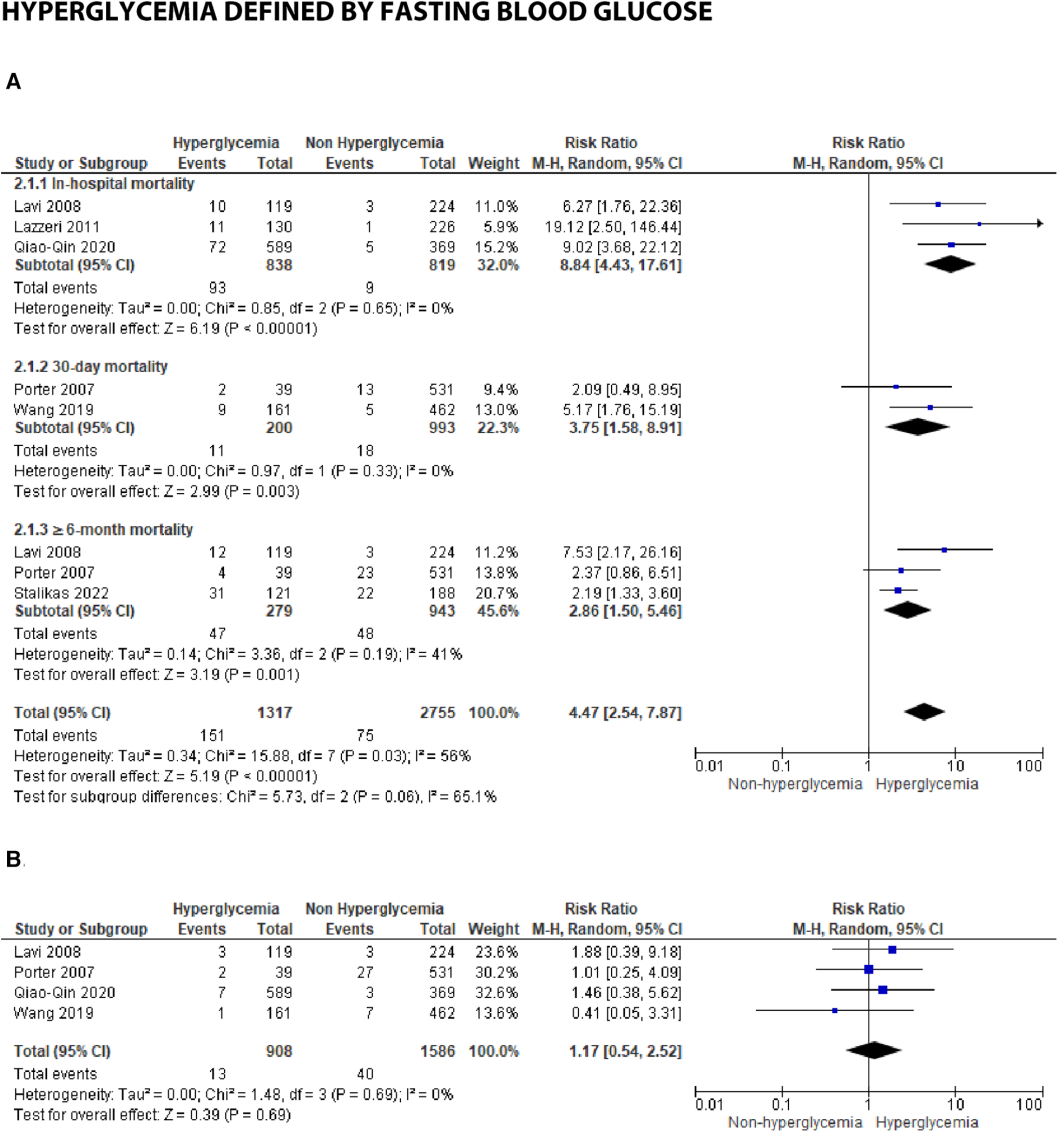
Forest plot of studies using fasting blood glucose as a parameter for hyperglycemia with outcomes of (**A**) mortality (in-hospital, 30 days, and ≥6 months), and (**B**) reinfarction or recurrent MI.

**Figure 3C:**
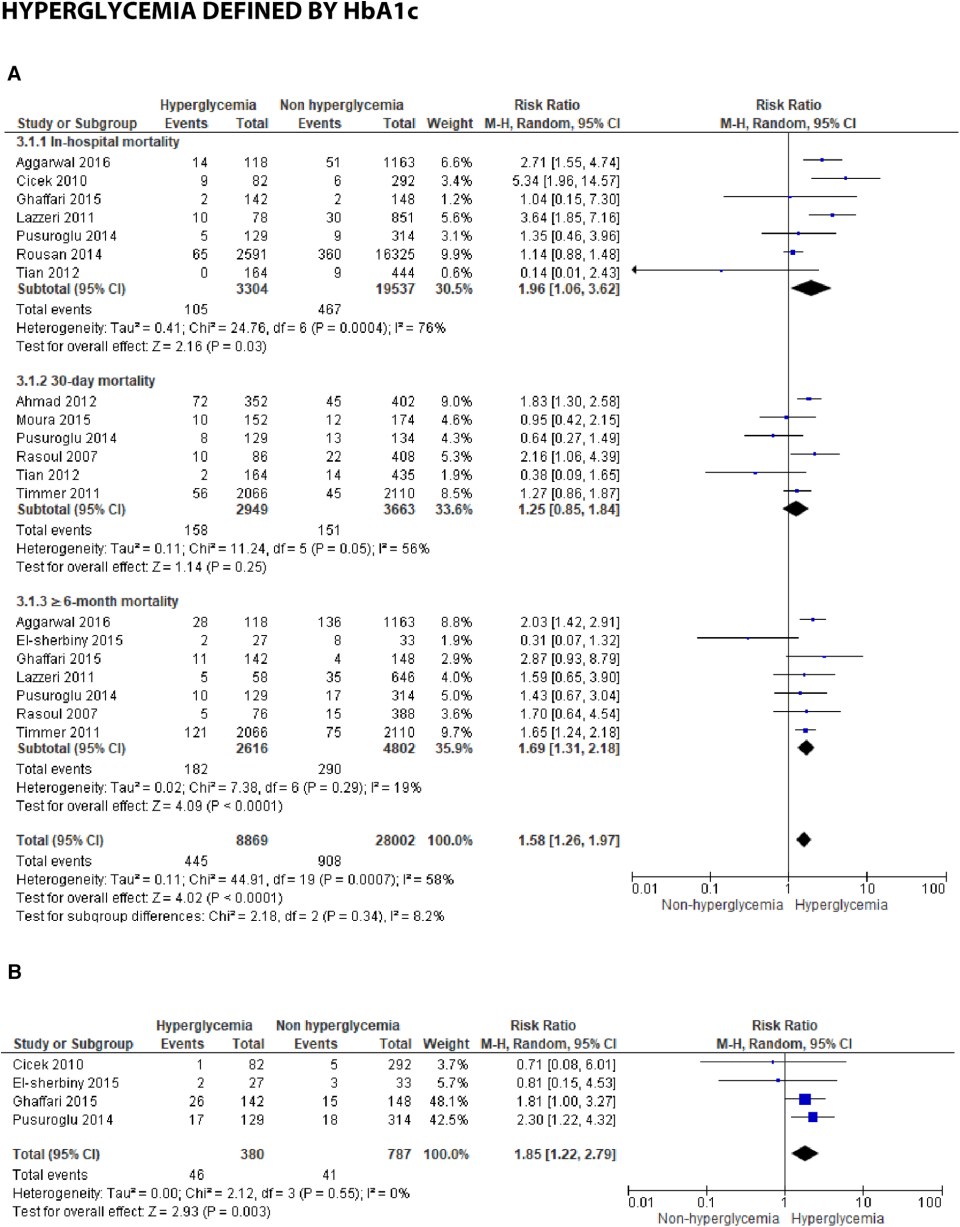
Forest plot of studies using HbA1c as a parameter for hyperglycemia with outcomes of (**A**) mortality (in-hospital, 30 days, and ≥6 months), and (**B**) reinfarction or recurrent MI.

### Hyperglycemia defined by admission blood glucose

(A) Mortality

Mortality outcomes in STEMI patients, using admission blood glucose as the predictor, were categorized into subgroups based on the time frame. Twenty-nine included studies assessing in-hospital mortality outcomes revealed a pooled risk ratio of 3.41 (2.82–4.14), underscoring the association between elevated admission blood glucose levels and the risk of in-hospital mortality. However, there was significant heterogeneity among studies in this subgroup. For 30-day mortality, 14 studies were included, yielding a pooled risk ratio of 3.71 (2.95–4.66) with *I*^2 ^= 86% indicating substantial heterogeneity among these studies. Similarly, for 6-month and >6-month mortality, both displayed consistent pooled risk ratios exceeding 1 with heterogeneity ranging between 66%–70%. Consequently, admission blood glucose exhibited a significant association with mortality across all time frames. However, when subjected to subgroup analysis, we identified an *I*^2^ value of 79.0% with *p* = 0.003, indicating considerable heterogeneity.

(B) Recurrent MI

The pooled risk ratio for recurrent MI and admission blood glucose levels demonstrated a statistically significant result of 1.69 (1.31, 2.17) with *I*^2 ^= 27% (*p* = 0.19), indicating only slight heterogeneity among the included studies.

(C) Heart failure

In the case of heart failure, admission blood glucose also exhibited a pooled risk ratio of 1.56 (1.37, 1.77) with *I*^2 ^= 0% (*p* = 0.55) and a Z-score for overall effect of 6.61 (*p* < 0.001). This outcome indicates a robust association between admission blood glucose levels and heart failure within the homogeneous STEMI population.

(D) Stroke

There was no observed association between stroke and admission blood glucose levels, as indicated by the pooled risk ratio of 1.50 (0.90, 2.49) and the overall effect (*Z* = 1.55, *p* = 0.12). The total combined events from both groups amounted to only 109.

(E) Cardiogenic shock

A pooled RR of 3.68 (2.65, 5.11) was identified for cardiogenic shock following STEMI. However, a notable level of heterogeneity was observed among the included studies, with *I*^2^ of 73%.

(F) Repeat PCI

The likelihood of requiring repeat percutaneous coronary intervention (PCI) was higher within the slightly heterogeneous STEMI population with elevated admission blood glucose levels. This was evident from the analysis of this outcome subgroup, which yielded a pooled RR of 1.99 (1.21, 3.28).

(G) Composite MACCE

Nine of the included studies combined adverse effects following STEMI into the MACCE group. It was determined that MACCE and admission blood glucose were significantly correlated, with a total pooled RR of 1.99 (1.545, 2.58), and a heterogeneity level of *I*^2 ^= 78% (*p* < 0.001).

(H) Emergency CABG

Two of the included studies analyzed emergency CABG as the outcome of interest. Admission blood glucose was shown to be not correlated emergency CABG, with a total pooled RR of 1.22 (0.46, 3.23), and a heterogeneity level of *I*^2^ = 0% (*p* = 0.99).

### Hyperglycemia defined by fasting blood glucose

Seven studies reported fasting blood glucose (FBG) as their parameter. Further analysis using meta-analysis explored the association between fasting blood glucose and in-hospital, 30-day, and ≥6-month mortality, as well as recurrent myocardial infarction.

(A) Mortality

Three studies were included in the subgroup assessing in-hospital mortality. The calculated pooled RR was 8.84 (4.43, 17.61) with minimal heterogeneity (*I*^2 ^= 0%, *p* = 0.65). Despite the smaller number of patients and studies compared to admission blood glucose, the risk ratio between FBG and in-hospital mortality was higher, approximately 2–3 times that of admission blood glucose's risk.

Similarly, the pooled risk ratios in the 30-day and ≥6-month mortality subgroups were higher compared to admission blood glucose, as depicted in the forest plot [30-days: 3.75 (1.58, 8.91) and ≥6 months: 2.86 (1.50, 5.46)]. Heterogeneity among the included studies in these subgroups was lower when compared to the same groups using admission blood glucose as the parameter (*I*^2 ^= 0%–41%).

(B) Reinfarction or recurrent MI

No significant association was found between FBG and recurrent myocardial infarction, with a total pooled RR of 1.17 (0.54, 2.52). Only four studies reported recurrent myocardial infarction for this blood glucose parameter, and low heterogeneity was observed among these studies (*I*^2 ^= 0%, *p* = 0.69.)

### Hyperglycemia defined by HbA1c

(A) Mortality

In studies employing HbA1c, several time points were reported, including in-hospital, 30-day, and ≥6-month mortality. An association was identified between HbA1c and in-hospital mortality, with a pooled RR of 1.96 (1.06, 3.62). However, the test revealed substantial heterogeneity, with an *I*^2 ^= 76% (*p* = 0.0004), among the included studies. Additionally, for 30-day mortality, the result was insignificant with a pooled RR of 1.25 (0.85, 1.84) and high heterogeneity (*I*^2 ^= 56%, *p* = 0.05). Furthermore, a significant association was found between increased HbA1c and mortality over 6 months post-STEMI, with a pooled RR of 1.69 (1.31, 2.18) and low heterogeneity (*I*^2 ^= 19%, *p* = 0.0007) among the studies. When analyzing the overall pooled risk ratio, HbA1c demonstrated a statistically significant association with overall mortality, with an RR of 1.58 (1.26, 1.97). Subgroup difference testing reported no difference between different mortality periods (*I*^2 ^= 8.2%, *p* = 0.34).

(B) ≥6-month recurrent myocardial infarction

An association was identified between the incidence of ≥6-month reinfarction and HbA1c, with a pooled risk ratio 1.85 (1.22, 2.79), within the homogenous studies (*I*^2 ^= 0%, *p* = 0.55).

#### Publication bias

**Figure 4:**
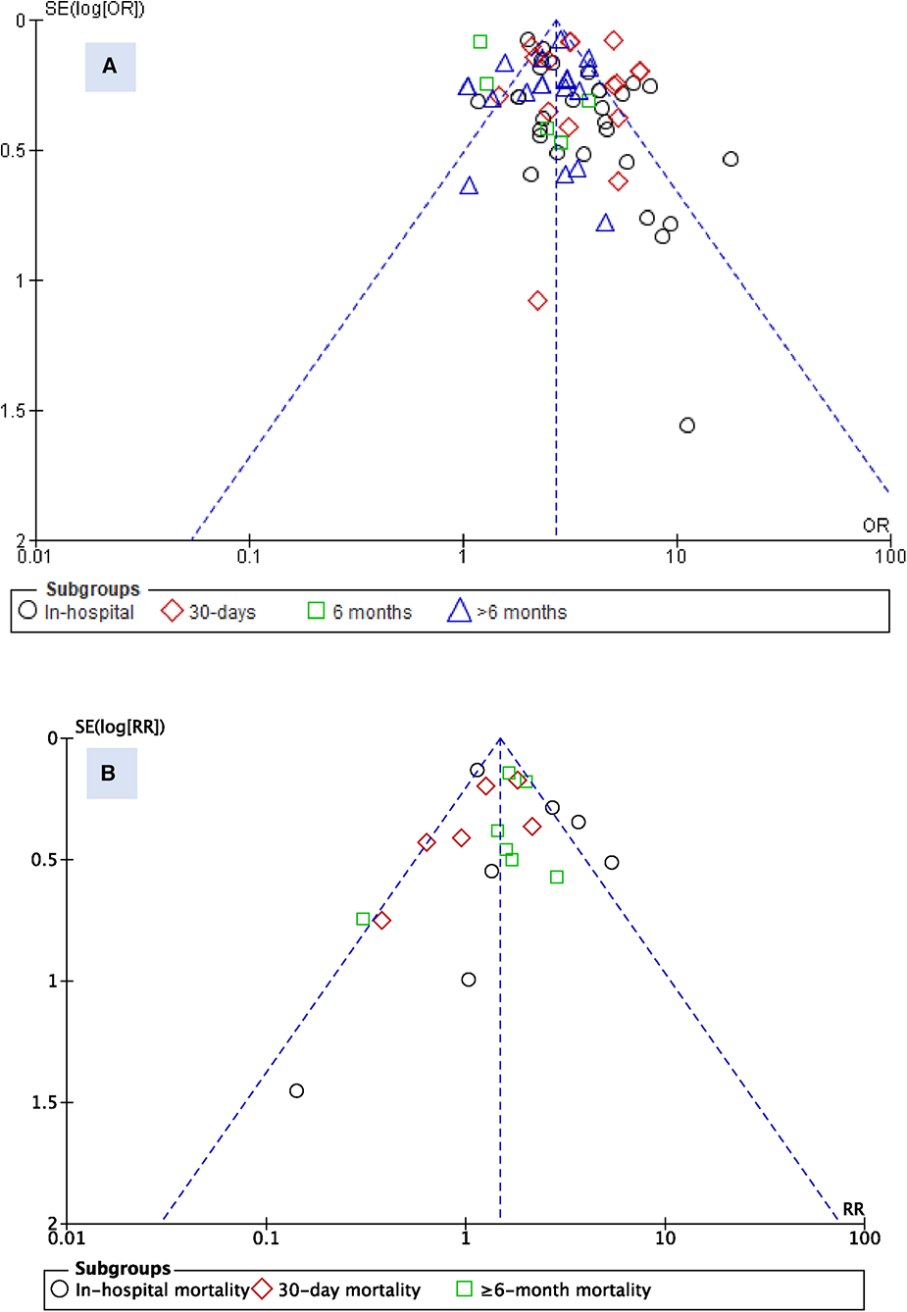
Funnel plot for studies reporting admission blood glucose (**A**) and HbA1c (**B**).

To assess for publication bias between studies, a funnel plot was used for visual assessment. While there is a probability of underreporting of studies with lower risk ratios, no substantial asymmetry was observed for the admission blood glucose parameter (Figure 4). However, for HbA1c, underreporting of studies with higher risk ratios was probable, particularly in the in-hospital mortality subgroup.

#### Summary of admission blood glucose's predicting accuracy

**Figure 5:**
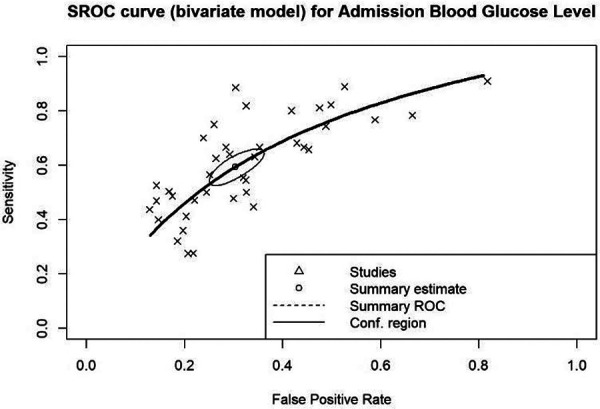
Summary receiver operating characteristics (ROC) curve for overall admission blood glucose levels (AUC: 0.688).

Efforts were made to determine the optimal cut-off for estimating mortality outcomes. The cut-off values used in the included studies to diagnose hyperglycemia within the populations were applied. It was discovered from the summary ROC curve that the AUC for admission blood glucose is 0.688, indicating that admission blood glucose is a reliable predictor for mortality outcome. The summary estimate reveals a sensitivity of 0.594 and a 1-specificity of 0.304, signifying that when using the optimal blood glucose cut-offs included, there is a 59.4% chance of correctly predicting outcomes in those with hyperglycemia, but a 30.4% tendency to incorrectly estimate the probability of those outcomes (Figure 5).

## Discussion

### Summary of main findings

After conducting a comprehensive review of the full-text articles, we identified 47 studies that utilized admission blood glucose, 13 studies that relied on HbA1c, and 7 articles that examined fasting blood glucose. The majority of the studies focused on primary PCI as their inclusion criteria, making it outside the scope of this systematic review to compare clinical outcomes between patients who underwent primary PCI and those who received optimal medical treatment. The most commonly reported outcomes related to hyperglycemia were in-hospital and 30-day mortality. Both admission and fasting blood glucose levels were significantly associated with in-hospital and short-term mortality following STEMI.

Among the 164,927 STEMI patients included, hyperglycemia upon admission was significantly related to all-cause mortality, irrespective of the follow-up period, with an RR of 3.02 (95%CI: 2.65–3.45). A more detailed analysis revealed that admission hyperglycemia independently predicted all-cause mortality, including in-hospital (RR 3.41, 95% CI: 2.82–4.14), at 30 days (RR 3.71, 95% CI: 2.95–4.66), at 6 months (RR 1.80, 95% CI: 0.96–3.36), and over 6 months of follow-up (RR 2.32, 95% CI: 1.90–2.82). These findings align with a previous systematic review by Capes et al. [NO_PRINTED_FORM] confirming the significant impact of admission blood glucose levels on short-term outcomes in myocardial infarction patients. Notably, even in non-diabetic patients, higher HbA1c levels were associated with increased mortality (74).

In this meta-analysis, we explored the association between hyperglycemia, as indicated by three clinically relevant parameters (i.e., admission, fasting, and Hb1Ac) and a broader range of clinical outcomes, some of which have not been previously reported in a single systematic review. We observed a significant relation between admission hyperglycemia and other short-term outcomes, including reinfarction, heart failure, cardiogenic shock, repeat PCI, and composite MACCE. However, our analysis did not find a significant link between admission hyperglycemia and stroke.

Utilizing FBG as a parameter of hyperglycemia showed similar effects on short-term outcomes, but no significant association was found between FBG and recurrent MI at 6 months. Our findings align with Li et al.'s previous review, which suggested that admission blood glucose levels are influenced by both acute physiological stress and chronic baseline glycemic levels, particularly in patients with known diabetes mellitus (21). We agree with this assessment, as our results using HbA1c as an indicator of acute hyperglycemia showed a significant association between hyperglycemia and mortality over a 6-month period, with a pooled RR of 1.69 (95% CI: 1.31–2.18). However, this association was not observed in short-term outcomes.

We conducted a subgroup analysis to examine the association between admission blood glucose and mortality, stratifying patients into those with and without DM. Our supplementary analysis revealed an elevated risk of mortality in both patient groups when presenting with hyperglycemia on admission, specifically observing a 46% increase in patients with DM and a 71% increase in patients without DM (Supplementary Figures S2,S3). Notably, the heightened mortality risk in patients with DM was observed primarily in the short term, but not in the longer observation term (>30-days). Numerous studies incorporated into our systematic review have indicated that, following multivariable analysis, elevated admission blood glucose levels in patients without DM emerged as an independent predictor of mortality. Conversely, this independent predictive association was not observed in patients with DM (18, 29, 34, 72). Direct comparison between DM vs. non-DM in STEMI patients who suffered from stresss hyperglycemia is shown in Supplementary Figure S4.

Noteworthy among these findings is the study conducted by Eitel et al. (22), which identified a correlation between higher myocardial injury and patients without DM. Additionally, the investigation by Ferreira et al (27) highlighted a noteworthy shift in the association between admission blood glucose levels and mortality risk among patients without DM, aligning more closely with those observed in patients with DM during long-term observations. In their recent meta-analysis, Karakasis et al. (75) proposed that the stress hyperglycemia ratio (SHR) might serve as a superior predictor compared to individual hyperglycemia parameters in assessing the association with MACE and mortality, regardless of the time period and diabetes status.

While we acknowledge the potential advantages of SHR, single hyperglycemia parameters such as admission blood glucose levels, fasting blood glucose, and HbA1c hold widespread recognition and routine utilization in clinical practice. These established metrics are familiar to clinicians, seamlessly integrated into diagnostic and management processes for cardiovascular events like ST-elevation myocardial infarction (STEMI), and readily available in standardized clinical settings. Although SHR offers the potential for more robust analysis and longer-term decision-making, the use of single hyperglycemia predictors remains advantageous for rapid and efficient clinical decisions in time-sensitive situations. This nuanced approach allows for a balanced integration of both methodologies, catering to the diverse needs of clinical practice.

#### Defining hyperglycemia and the cutoff level

Although numerous studies on stress hyperglycemia in the context of myocardial infarction have been published, there is currently no universally accepted definition for stress hyperglycemia in this setting. Previous studies has often relied on admission blood glucose for defining hyperglycemia (2, 3, 14–18). The most widely accepted definition for admission blood glucose is the first collected blood glucose measurement within 24 h of hospital admission. However, the specific cutoff point used to define hyperglycemia in STEMI patients has varied among previous studies.

In 2008, the American Heart Association (AHA) Scientific Statement on Hyperglycemia and Acute Coronary Syndrome suggested using a cutoff point of >140 mg/dl for admission blood glucose levels to define hyperglycemia in such cases (76). A prior meta-analysis by Capes et al. indicated that among non-diabetic patients with acute myocardial infarction, those with an admission glucose level >110 mg/dl had a 3.9 times higher relative risk of in-hospital mortality compared with the normoglycemic patients. Additionally, among diabetic patients with acute myocardial infarction, an increased risk of in-hospital mortality was observed only in those with admission glucose levels ≥180 mg/dl (74). Despite several studies investigating the effects of hyperglycemia on cardiovascular outcomes, a consensus on the definition or cut-off for acute hyperglycemia in myocardial infarction patients has not been reached (5).

In this systematic review, the included studies employed various cutoff points for admission (random) blood glucose: 19 studies used a level of ≥198 mg/dl (11.0 mmol/L); 7 studies used ≥180 mg/dl (10 mmol/L); 13 studies used ≥140 mg/dl (7.8 mmol/L); 2 studies used ≥126 mg/dl (7 mmol/L), and 1 study used cutoff >110 mg/dl. Additionally, 7 studies used a cutoff of ≥126 (7 mmol/L) for FBG. For the parameter of HbA1c, 6 studies employed a cut off of ≥6.5%, and 1 study used a cutoff of ≥5.5%. The selection of cutoff values for admission hyperglycemia is in line with the guidelines provided by the American Diabetes Association (ADA), which recommends initiating insulin therapy for persistent hyperglycemia >180 mg/dl (10 mmol/L) (74).

Based on these findings, we suggest adopting a lower cutoff point of ≥140 mg/dl (7.8 mmol/L) for non-diabetic patients as the definition of admission blood glucose hyperglycemia. For diabetic patients, a higher cutoff point of ≥180 mg/dl (10 mmol/L) could be considered. In the case of FBG, we recommend using a cutoff point of ≥126 mg/dl (7 mmol/L) to quantify stress hyperglycemia.

The variation in cutoff values for admission glucose and their predictive accuracy for adverse outcomes between diabetic and non-diabetic patients with acute myocardial infarction may be attributed to differences in baseline glucose metabolic status. Admission glucose levels are influenced by both acute physiological stress and chronic baseline glycemic levels, particularly in patients with established diabetes mellitus. Several criteria were used in this review to define diabetes mellitus, including fasting blood glucose ≥126 mg/dl (7 mmol/L), 2-h post-prandial blood glucose ≥200 mg/dl (11.1 mmol/L), HbA1c ≥ 6.5%, and classic symptoms of hyperglycemia with random blood glucose ≥200 mg/dl (11.1 mmol/L), or a self-reported history of diabetes or the use of hypoglycemic agents.

#### Pathophysiology of stress-induced hyperglycemia in STEMI

Hyperglycemia is a frequent response in patients with STEMI, even among those without a previous history of diabetes mellitus (77). Several mechanisms underlie the occurrence of hyperglycemia in myocardial infarction patients. The primary mechanism involves the activation of the sympathetic nervous system and heightened activity of the hypothalamic-pituitary axis, leading to the production of catecholamines and glucocorticoids, in the form of cortisol (78). Additionally, increased sympathetic nerve activity triggers the release of glucagon, which stimulates glycogenolysis in muscles and the liver, resulting in the breakdown of glycogen into glucose that enters the circulation (79, 80).

**Figure 6:**
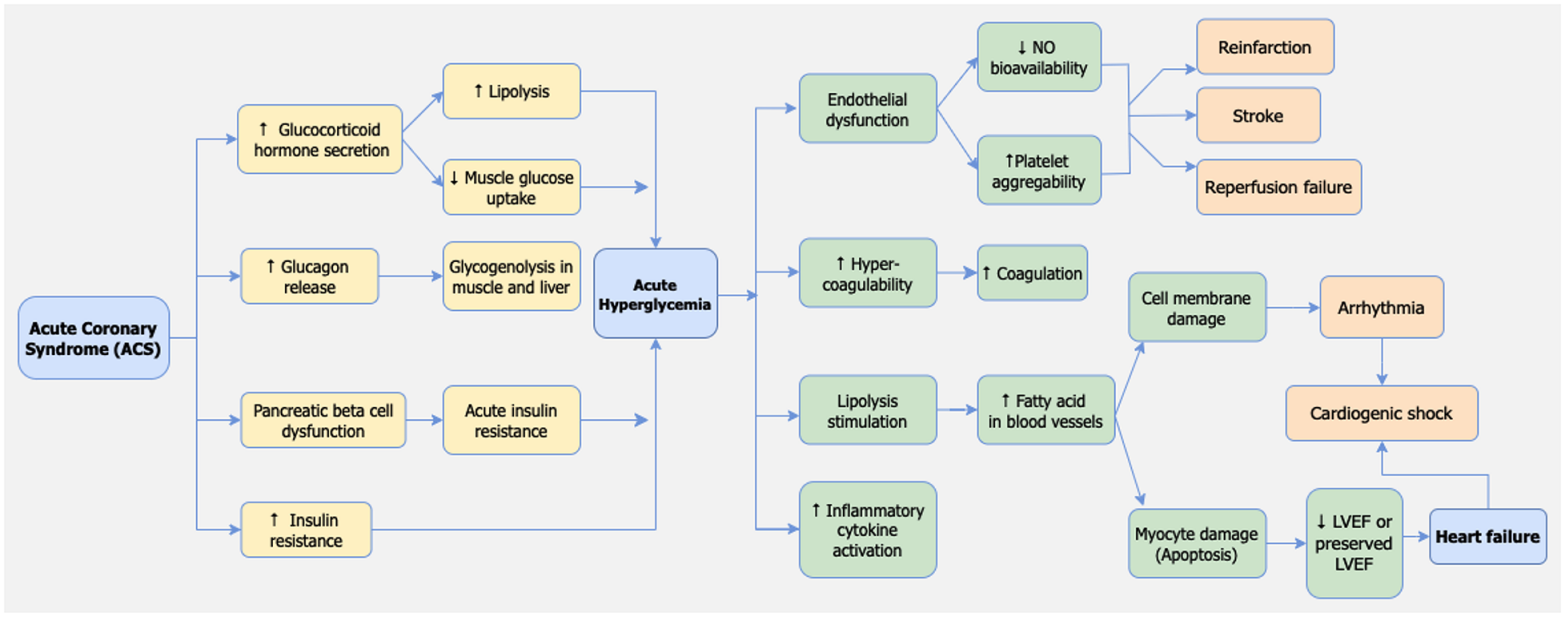
Pathophysiology of adverse events caused by stress hyperglycemia in STEMI patients.

Other contributing mechanisms include dysfunction of pancreatic beta cells and insulin resistance due to acute stress conditions. The precise pathophysiology of these mechanisms remains unclear (78). However, previous studies have indicated a link between acute stress conditions during myocardial infarction and beta cell dysfunction, leading to reduced insulin production and increased insulin resistance. It is possible that hyperglycemia is merely an expression of stress and a consequence of the acute-phase reaction in the clinical setting of acute myocardial infarction. This reaction involves a series of neurohormonal responses activated during MI, leading to the overactivation of sympathetic nerves. Concurrently, the activation of stress-induced cortisol, noradrenaline, growth hormone, and glucagon release can disrupt glucose homeostasis and insulin secretion, resulting in insulin insufficiency and acute hyperglycemia. This mechanism is illustrated in Figure 6.

#### Mechanism on how hyperglycemia adversely affect the STEMI outcomes

Hyperglycemia is a common occurrence during the early stages of STEMI, even in patients with no previous history of diabetes mellitus. Increased sympatho-adrenergic activation and dysregulation of adrenergic receptors, such as beta-adrenergic receptors modulated by G protein coupled receptor kinase (GRK) (81), can contribute to various complications, including heart failure and arrhythmias (9).

Several mechanisms have been proposed to explain why hyperglycemia serves as a significant predictor of poor outcomes in STEMI patients, including arrhythmias, heart failure, and hospital death. First, Pres et al. reported that acute hyperglycemia in STEMI patients leads to vascular endothelial dysfunction (51). Similar findings were observed by Dong Bao et al. who demonstrated that endothelial dysfunction in the presence of acute hyperglycemia reduces the bioavailability of Nitric oxide (NO) and increases platelet aggregability. These effects can contribute to reperfusion failure in STEMI patients with acute hyperglycemia (21). Second, Kocas et al. reported that hyperglycemia in STEMI patients causes an increase in prothrombin fragments and factor VII levels while inhibiting tissue plasminogen activator. This leads to blood hypercoagulability in patients with hyperglycemia, further contributing to reperfusion failure (5). Third, hyperglycemia may reflect relative insulin deficiency, resulting in increased lipolysis and elevated levels of circulating free fatty acids. Pres et al. reported that hyperglycemia stimulates lipolysis, thereby increasing free fatty acid in blood flow. This increase in free fatty acids in the bloodstream can be toxic to the ischemic or infarcted myocardium, exacerbating myocyte injury, calcium overload, and arrhythmias. Insulin deficiency also reduces the myocardium's ability to utilize glucose anaerobically (51).

Furthermore, hyperglycemia-induced damage to the myocardium can lead to contractility disorders, adversely affecting the already ischemic or infarcted myocardium and reducing the left ventricular ejection fraction (LVEF) (51). In addition, Chen et al. reported that hyperglycemia is associated with increased activity of inflammatory cytokines, contributing to damage in heart muscle cells (Figure 6) (3).

Paradoxically, Kocas et al. reported that hypoglycemia in STEMI patients could also increase the risk of death. This suggests a U-shaped correlation between blood glucose levels and STEMI outcomes, where both hyperglycemia and hypoglycemia are associated with poor outcomes (5). This is supported by the study by Ishihara et al. who reported that hypoglycemia would adversely impact on STEMI patients outcomes. There are several factors contributing to hypoglycemia in STEMI patients, including the effects of anti-diabetic medications, hepatic gluconeogenesis dysfunction, and relative adrenal insufficiency (82). However, it's important to note that in our systematic review, we specifically focused on examining the relationship between hyperglycemia and outcomes in STEMI patients.

**Figure 7:**
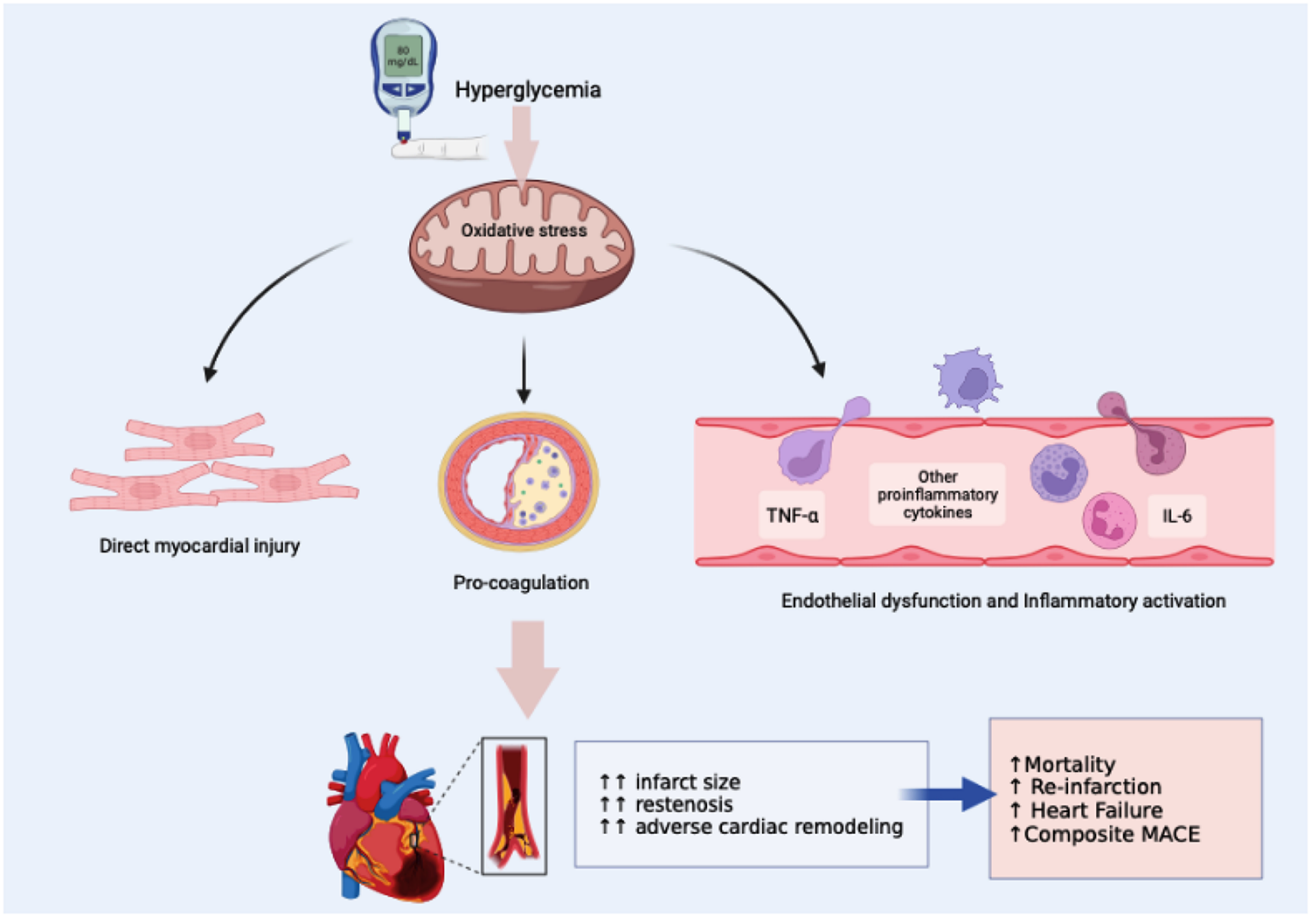
Mechanisms underlying the adverse effects of hyperglycemia in STEMI (figure modified from Li et al. (83)).

**﻿** In experimental models, hyperglycemia has been associated with various detrimental effects. It leads to increased expression of adhesion molecules, enhances leukocyte adhesion to capillary walls, exacerbates free-radical-related reperfusion injury, and induces excessive apoptosis of endothelial cells. Furthermore, hyperglycemia augments the formation of microthrombi by increasing platelet aggregation and elevates circulating cytokine levels (including IL-6, IL-18, TNF- *α*, and CCL-2) contributing to instability in atheromatous plaques. Hyperglycemia may also disrupt endothelium-dependent vasodilatation and impair endogenous fibrinolysis (as depicted in Figure 7) (84). In addition to stress-induced hyperglycemia, long-lasting hyperglycemia, primarily stemming from underlying glucose metabolism abnormalities, can play a significant role in the progression of atherosclerosis, potentially leading to more extensive coronary artery disease. This persistent hyperglycemia can result in diffuse endothelial dysfunction, affecting both diastolic and systolic function in addition to the infarcted myocardium (85).

Hyperglycemia has secondary effects that include prothrombotic and proinflammatory effects. It can lead to the formation of thrombi in microvasculature, potentially accelerating remodeling of the surrounding myocardium while impeding collateral vascular development. Additionally, the recovery of stunned myocardium may be more challenging in a hyperglycemic environment, as hyperglycemia has been shown to be associated with several markers indicative of left ventricular dysfunction (85). Acute hyperglycemia aggravates platelet-dependent thrombus formation, attenuates endothelium-dependent vasodilatation, and reduces collateral blood flow by negatively affecting nitric oxide availability. These changes are linked to microvascular dysfunction before reperfusion and could influence the outcomes of interventions.

The impact of managing hyperglycemia on long-term patient outcomes has been explored in various studies. Rigorous glycemic control has demonstrated its effectiveness in reducing multiple organ failure, infection, and mortality both in the short and long term (86). However, navigating the complexities of hyperglycemia management in hospitalized patients, including those with diabetes, is intricate, and the treatments effect on long-term outcomes is influenced by various factors. For instance, findings from the Stroke Hyperglycemia Insulin Network Effort (SHINE) trial revealed that, among patients with acute ischemic stroke and hyperglycemia, the difference in functional outcomes between intensive and standard glucose control for up to 72 h was not statistically significant (87). Additionally, evidence from the The Diabetes Control and Complications Trial (DCCT), Epidemiology of Diabetes Interventions and Complications (EDIC), and the UK Prospective Diabetes Study (UKPDS) cohorts suggested that early control of hyperglycemia in recently diagnosed diabetes has a lasting impact on long-term outcomes (88). Hence, while intense glycemic control has demonstrated advantages in certain studies, the intricacies of hyperglycemia management and its implications for long-term outcomes represent a complex and evolving area of research.

To our knowledge, this is the first review to utilize a comprehensive meta-analysis to assess various clinical outcomes in STEMI patients. The interpretations derived from this review can generally apply to diverse populations as we collected studies from various countries. In this systematic review, we did not impose a fixed timeframe for measuring secondary outcomes such as reinfarction, recurrent MI, incidence of HF, cardiogenic shock, and stroke. The included studies reported varied time points from short-term (in-hospital and 30-day) to long-term (≥6 months), which potentially introducing variability and heterogeneity into the estimated relative risk values.

In conclusion, hyperglycemia has a strong association with adverse outcomes following STEMI. Admission and FBG levels may serve as predictors for short-term outcomes in STEMI patients, whereas HbA1c may be a more suitable indicator for forecasting longer-term outcomes.

## Data Availability

The original contributions presented in the study are included in the article/**Supplementary Material**, further inquiries can be directed to the corresponding author.
