# Supplementary material for: Stress hyperglycemia and poor outcomes in patients with ST-elevation myocardial infarction: a systematic review and meta-analysis
Source: Front Cardiovasc Med. 2024 Mar 11;11:1303685. doi: 10.3389/fcvm.2024.1303685 (PMC10961461; doi:10.3389/fcvm.2024.1303685)

# **Supplementary materials:**

**Table S1.** Search strategy for Pubmed and Embase

| **PUBMED** | |
| --- | --- |
| #1 | ("hyperglycaemia"[Title/Abstract] OR "hyperglycemia"[MeSH Terms] OR "hyperglycemia"[Title/Abstract] OR "hyperglycaemias"[Title/Abstract] OR "hyperglycemias"[Title/Abstract] OR "hyperglycemia s"[Title/Abstract]) OR ("glucose"[Text Word] OR "FPG"[Text Word] OR "plasma glucose"[Text Word] OR "blood glucose"[Text Word]) |
| #2 | "st elevation myocardial infarction"[MeSH Terms] OR ("myocardial"[Title/Abstract] AND "infarction"[Title/Abstract]) OR "st elevation myocardial infarction"[Title/Abstract] OR ("segment"[Title/Abstract] AND ("elevation"[Title/Abstract] OR "elevated"[Title/Abstract]) AND "myocardial"[Title/Abstract] AND "infarction"[Title/Abstract]) OR "st segment elevation myocardial infarction"[Title/Abstract] OR "Acute coronary syndrome"[Title/Abstract] OR "acute coronary syndromes"[Title/Abstract] OR "coronary syndrome"[Title/Abstract] OR (("acute myocardial infarction"[Title/Abstract] OR "AMI"[Title/Abstract]) AND ("percutaneous coronary intervention"[Title/Abstract] OR "PCI"[Title/Abstract]) OR "STEMI"[Title/Abstract] OR "ACS"[Title/Abstract] |
| #3 | "prognosis"[MeSH Terms] OR "prognosis"[Title/Abstract] OR "prognoses"[Title/Abstract] OR ("outcome"[Title/Abstract] OR "outcomes"[Title/Abstract]) OR (("adverse"[Title/Abstract] OR "adversely"[Title/Abstract] OR "adverses"[Title/Abstract]) AND ("event"[Title/Abstract] OR "event s"[Title/Abstract] OR "events"[Title/Abstract])) OR ((("major"[Title/Abstract] OR "majored"[Title/Abstract] OR "majoring"[Title/Abstract] OR "majorities"[Title/Abstract] OR "majority"[Title/Abstract] OR "majors"[Title/Abstract]) AND ("cardiovascular system"[MeSH Terms] OR ("cardiovascular"[Title/Abstract] AND "system"[Title/Abstract]) OR "cardiovascular system"[Title/Abstract] OR "cardiovascular"[Title/Abstract] OR "cardiovasculars"[Title/Abstract]) AND ("adverse"[Title/Abstract] OR "adversely"[Title/Abstract] OR "adverses"[Title/Abstract]) AND ("event"[Title/Abstract] OR "event s"[Title/Abstract] OR "events"[Title/Abstract])) OR "mace"[Title/Abstract]) OR ("macce"[Title/Abstract] OR "macces"[Title/Abstract]) OR ("mortality"[MeSH Terms] OR "mortality"[Title/Abstract] OR "mortalities"[Title/Abstract] OR "mortality"[MeSH Subheading]) OR ("death"[MeSH Terms] OR "death"[Title/Abstract] OR "deaths"[Title/Abstract]) OR (("cardiovascular system"[MeSH Terms] OR ("cardiovascular"[Title/Abstract] AND "system"[Title/Abstract]) OR "cardiovascular system"[Title/Abstract] OR "cardiovascular"[Title/Abstract] OR "cardiovasculars"[Title/Abstract]) AND ("mortality"[MeSH Terms] OR "mortality"[Title/Abstract] OR "mortalities"[Title/Abstract] OR "mortality"[MeSH Subheading])) OR (("cardiovascular system"[MeSH Terms] OR ("cardiovascular"[Title/Abstract] AND "system"[Title/Abstract]) OR "cardiovascular system"[Title/Abstract] OR "cardiovascular"[Title/Abstract] OR "cardiovasculars"[Title/Abstract]) AND ("death"[MeSH Terms] OR "death"[Title/Abstract] OR "deaths"[Title/Abstract])) OR ("rehospitalization"[Title/Abstract] OR "rehospitalizations"[Title/Abstract] OR "rehospitalized"[Title/Abstract]) OR ("recurrance"[Title/Abstract] OR "recurrence"[MeSH Terms] OR "recurrence"[Title/Abstract] OR "recurrences"[Title/Abstract] OR "recurrencies"[Title/Abstract] OR "recurrency"[Title/Abstract] OR "recurrent"[Title/Abstract] OR "recurrently"[Title/Abstract] OR "recurrents"[Title/Abstract]) OR ("reinfarct"[Title/Abstract] OR "reinfarction"[Title/Abstract] OR "reinfarctions"[Title/Abstract]) OR (("stent s"[Title/Abstract] OR "stentings"[Title/Abstract] OR "stents"[MeSH Terms] OR "stents"[Title/Abstract] OR "stent"[Title/Abstract] OR "stented"[Title/Abstract] OR "stenting"[Title/Abstract]) AND ("thrombose"[Title/Abstract] OR "thrombosing"[Title/Abstract] OR "thrombosis"[MeSH Terms] OR "thrombosis"[Title/Abstract] OR "thrombosed"[Title/Abstract] OR "thromboses"[Title/Abstract])) OR (("stent s"[Title/Abstract] OR "stentings"[Title/Abstract] OR "stents"[MeSH Terms] OR "stents"[Title/Abstract] OR "stent"[Title/Abstract] OR "stented"[Title/Abstract] OR "stenting"[Title/Abstract]) AND ("blockage"[Title/Abstract] OR "blockages"[Title/Abstract])) OR (("stent s"[Title/Abstract] OR "stentings"[Title/Abstract] OR "stents"[MeSH Terms] OR "stents"[Title/Abstract] OR "stent"[Title/Abstract] OR "stented"[Title/Abstract] OR "stenting"[Title/Abstract]) AND ("embolis"[Title/Abstract] OR "embolism"[MeSH Terms] OR "embolism"[Title/Abstract] OR "emboli"[Title/Abstract])) OR (("stent s"[Title/Abstract] OR "stentings"[Title/Abstract] OR "stents"[MeSH Terms] OR "stents"[Title/Abstract] OR "stent"[Title/Abstract] OR "stented"[Title/Abstract] OR "stenting"[Title/Abstract]) AND "clot"[Title/Abstract]) OR ("thrombosis"[MeSH Terms] OR "thrombosis"[Title/Abstract] OR ("blood"[Title/Abstract] AND "clot"[Title/Abstract]) OR "blood clot"[Title/Abstract]) OR ("repeat"[Title/Abstract] OR "repeating"[Title/Abstract] OR "repeats"[Title/Abstract]) OR "redo"[Title/Abstract] OR ("emerge"[Title/Abstract] OR "emerged"[Title/Abstract] OR "emergence"[Title/Abstract] OR "emergences"[Title/Abstract] OR "emergencies"[MeSH Terms] OR "emergencies"[Title/Abstract] OR "emergency"[Title/Abstract] OR "emergent"[Title/Abstract] OR "emergently"[Title/Abstract] OR "emergents"[Title/Abstract] OR "emerges"[Title/Abstract] OR "emerging"[Title/Abstract]) OR "compulsory"[Title/Abstract] OR ("urgent"[Title/Abstract] OR "urgently"[Title/Abstract]) OR poor[tiab] OR readmission[tiab] NOT ("Editorial"[Publication Type] OR "Letter"[Publication Type] OR "case reports"[Publication Type] OR "Comment"[Publication Type] OR "review"[Publication Type] OR "systematic review"[Publication Type] OR "meta-analysis"[Publication Type])) NOT ("animals"[MeSH Terms] NOT "humans"[MeSH Terms]) |
| #4 | (mmol/L OR mg/dl) |
| #5 | #1 AND #2 AND #3 AND #4 |

| **EMBASE** | |
| --- | --- |
| #1 | ((hyperglycemia OR fpg OR plasma) AND glucose OR blood) AND glucose |
| #2 | ((((((prognosis OR outcome OR adverse) AND event OR major) AND adverse AND cardiovascular AND event OR mace OR macce OR mortality OR death OR rehospitalization OR recurrence OR reinfarction OR stent) AND thrombose OR stent) AND blockage OR stent) AND emboli OR stent) AND blood AND clot OR repeat OR emergency OR compulsory OR urgent OR poor OR readmission |
| #3 | st AND elevat* AND myocard*l AND infarct* OR (acute AND coronary AND syndrome) OR ((acute AND myocard* AND infarct* OR (myocard* AND infarct*) OR ami) AND (percutaneous AND coronary AND intervention OR pci)) OR acs OR stemi |
| #4 | #1 AND #2 AND #3 |
| #5 | #1 AND #2 AND #3 AND ([article]/lim OR [conference paper]/lim OR [data papers]/lim OR [preprint]/lim) NOT 'case report' NOT 'systematic review' AND [humans]/lim AND [english]/lim AND [2022-2023]/py |

**Figure S1.** Risk of bias traffic-light plot of the included studies

# **
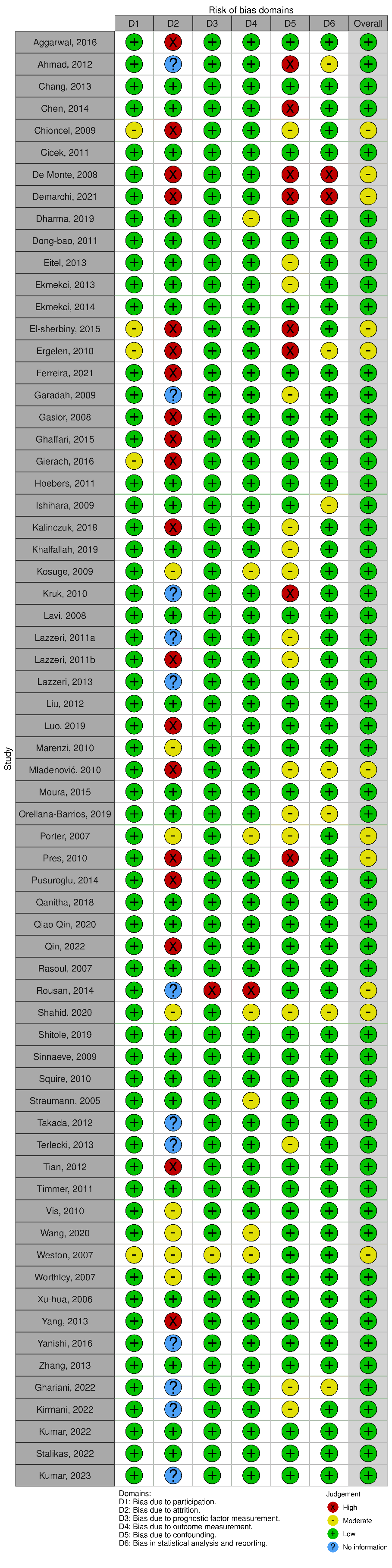
**
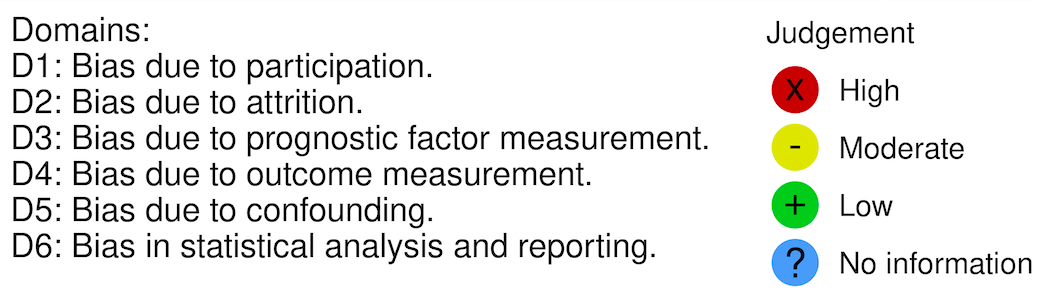


#

**Figure S2.** Forest plot of short-term mortality (inhospital to 30 days) and admission blood glucose in patients with DM


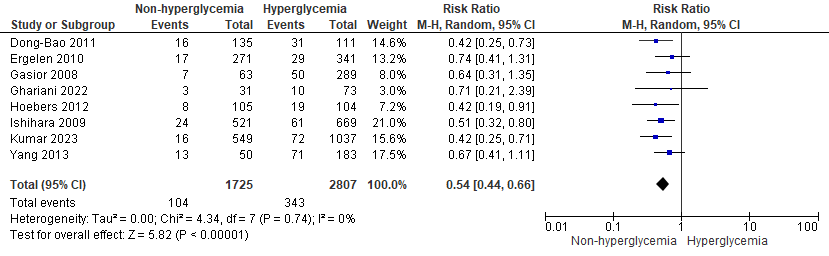


**Figure S3.** Forest plot of mortality and admission blood glucose in patients without DM


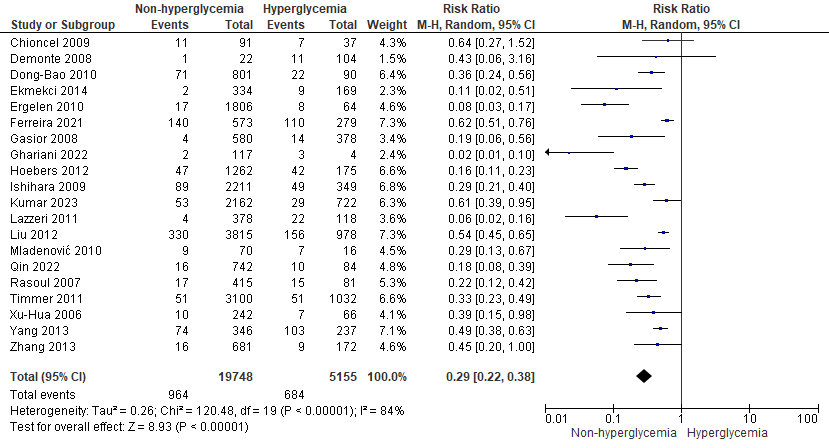


**Figure S4.** Forest plot of mortality and admission blood glucose in patients with DM compared to those without DM


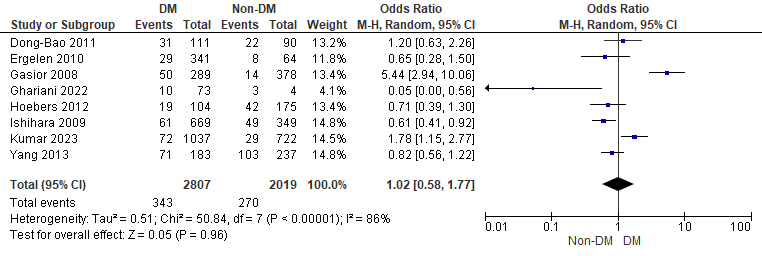

Supplement: Supplementary file 1 [file Table1.docx]
